# A novel smart hybrid multimorph piezoelectric spherical shell cloak for broadband near-perfect underwater acoustic camouflage applications

**DOI:** 10.1038/s41598-024-63201-w

**Published:** 2024-06-07

**Authors:** Seyyed M. Hasheminejad, Ali Kasaeisani

**Affiliations:** Acoustics Research Laboratory, Center of Excellence in Experimental Solid Mechanics and Dynamics, School of Mechanical Engineering, Iran University of Science and Technology, Narmak, Tehran, 16846-13114 Iran

**Keywords:** Smart hybrid multimorph actuation, Multi-stacked piezo-composite shell, Metamaterial-free acoustic cloaking, Broadband acoustic invisibility, Omnidirectional anti-sonar camouflage, Underwater acoustic target recognition, Energy science and technology, Engineering, Mathematics and computing, Physics

## Abstract

Non-visual auditory camouflage plays a major role in the art of underwater deception. In this work, a hybrid active/semi-active omnidirectional cloaking shell structure composed of alternate complementary piezoelectric and smart viscoelastic (PZT/SVE) actuator layers is proposed that can effectively conceal a three dimensional underwater macroscopic object from broadband incident sound waves. The smart hybrid structure incorporates a finite sequence of fully active parallel-connected multimorph PZT constraining layers inter-stacked with semi-active SVE core layers both of which are collaboratively operative in the framework of a Particle Swarm Optimized (PSO) multiple-input multiple-output active damping control (MIMO-ADC) scheme. The elasto-acoustic modeling of the problem is conducted by coupling the spatial state space methodology based on the classical three-dimensional exact piezoelasticity theory with the wave equations for the inner and outer acoustic domains. The acoustic cloaking performance of proposed configuration is evaluated for four distinct classes of highly functional SVE interlayer materials with tunable (field-dependent) rheological properties, namely, magnetorheological elastomer (MRE), shape memory polymer (SMP), electrorheological fluid (ERF), and magnetorheological shear thickening polishing fluid (MRSTPF). Extensive numerical results reveal significant broadband reductions of the far-field backscattering amplitude in the ($\left|{f}_{\infty }\left(\theta =\pi ,{k}_{\text{ex}}{R}_{\text{ex }}\right)\right|)$ as well as the percentage error of external cloaked field $(\%\text{Err})$ by incorporating a sufficient number of smart multimorph PZT/SVE material layers. Furthermore, it is concluded that comparable low frequency acoustic cloaking effects is possible without expenditure of any external energy just by employing the entirely inactive MRSTPF-based cloak as an alternative to the semiactive or fully active multimorph PZT/SVE cloaks. The outcome of proposed study can advantageously serve as the first step towards practical development and experimental implementation of future high performance smart acoustic cloaking devices with expanded broadband near-perfect omnidirectional invisibility for three dimensional objects of diverse geometries.

## Introduction

Underwater object recognition and classification based on target acoustic scattering is an attractive and challenging topic in field of underwater acoustics and sonar systems. The acoustic scattering characteristics of underwater objects are strongly correlated with the key object attributes such as size, material, and structure as well as with the target environment and incident wave field conditions. The earliest models studied the spherical shell as a standard quiet underwater structure due to its resemblance to many synthetic underwater targets and devices^1–4^. Analytical solutions to acoustic scattering and radiation from an isolated fluid-submerged elastic spherical shell that date back to the 1960s have provided a clear intuitive physical image of the problem^5–7^. The spherical configuration is also considered as a generic three dimensional acoustic cloaking device that can elegantly minimize or totally eradicate the acoustic traces of enclosed arbitrary-shaped three dimensional objects against incident sound waves^8–14^. In particular, the spherical cloak has the significant ability of concealing irregular objects from detection in any arbitrary incidence direction owing to the unique spherical symmetry (i.e., the so-called all-angle invisibility or omnidirectionality). Furthermore, in contrast to the 3D cloaking devices of non-canonical shapes (e.g., slab, cuboidal, columnar, elliptical, conical, pyramidal, tetrahedron, polygonal, and toroidal cloaks), the invisibilty cloaks of spherical shape are more suitable for realistic applications, as they are relatively easier to design and theoretical analyze. In this context, 3D acoustic wave field cloaking has been the subject of quite a few studies since the pioneering works of Refs^15–17^. Such systems are getting increasingly more refined as the detection methods become more sophisticated. Considerable attention has been recently paid to the cloaking systems that can offset the overall scattering behavior in a broad frequency range rather than systems that only treat particular aspects such as reduction of diffraction shadows and/or backscattering reflections.

The current cloaking literature includes three general categories, namely, the passive, active, and hybrid active–passive methods. The cloaking performance of passive methods, which do not require expenditure of external energy and/or expensive signal processing equipment, may be further improved through optimization of the scatterer geometrical and material properties as well as modification of the immediate surrounding and boundary. However, the physical properties of conventional engineering materials can substantially limit the cloaking performance particularly for high frequency incident waves. Therefore, the recent passive cloaking methodologies consider enclosing the scatterer with layers of exotic metamaterials that could include subwavelength geometrical structures with effective macro properties. Presently, there exist two main classes of passive cloaking schemes: coordinate transformation (CT) cloaking^18–21^ and scattering cancellation (SC) cloaking^22–24^. The CT-based cloaking, despite its sophisticated mathematical presentation, encounters some serious issues in regard to its practical implementation even with the profit of exotic metamaterials. Alternatively, the SC cloaking technique have recently been employed in which, rather than directly prohibiting the interactions with the incoming waves by detouring around the object, only the scattered waves are eliminated. On the other hand, as the passive CT- and SC-based cloaking designs are well-known to be inflexible with respect to the changes in the scattering object and the incident wave environment, the more adaptable and robust fully active or semi-active approaches that mainly rely on operation of sensors (microphones) and actuators (secondary control sources) in the framework of sophisticated measurement and control systems have recently been utilized to overcome such performance deficiencies at the expense of increased system complexity and power expenditure^25^. In what follows, an exhaustive survey of the subject is avoided and we shall briefly review the key contributions in the above mentioned three general categories (i.e., the passive CT- and SC- based cloaking, and active cloaking) with specific emphasis on the exterior acoustic cloaking configurations that are designed for camouflaging 2D or 3D objects. For recent detailed bibliographic surveys on the topic, the reader should consider Refs^18–21,26–28^.

Quite a few authors have adopted the CT-based approaches in the past few decades to design acoustic invisibility cloaks for camouflaging 2D or 3D objects from detection since the pioneering work of Pendry et al.^29^ on electromagnetic wave cloaking. For instance, Chen and Chan^15^ demonstrated perfect acoustic cloaking based on 3D mapping of the acoustic equation to the direct current conductivity equation and with the use of spherical-Bessel function series expansions. The invariance property of the acoustic equation was identified and a general transformation for the bulk modulus and density was obtained. Cummer and Schurig^17^ recognized an exact analogy between the 2D anisotropic electromagnetics and acoustics, and indicated that a 2D acoustic cloaking shell exists. However, they concluded that this isomorphism does not apply to 3D, and a 3D acoustic cloaking shell (if it exists) is nothing like its electromagnetic matching part. Cheng and Liu^30^ employed a layered acoustic system comprised of concentric multilayered spherical shells filled with homogeneous isotropic materials to design a spherical acoustic cloak for efficient broadband cloaking of a 3D object from detection in arbitrary direction. It was found that the designed structure could behave as an effective 3D transformation medium with each layer having proper bulk modulus and density. Scandrett et al.^31^ studied a wide range of cloaks from those comprised of fluid layers which are isotropic in bulk moduli with anisotropic density (pure inertial cloak) to those having anisotropic bulk moduli and isotropic density (pure pentamode cloak). Scandrett et al.^32^ subsequently introduced a novel methodology for finding optimal material parameters of pentamode-layered acoustic cloaking systems that can be effective in maximum bandwidth without unrealizable material properties within each layer. Torrent and Sánchez-Dehesa^33^ used homogenization of two interchanging isotropic fluid-like materials to design 2D imperfect acoustic cloaks with reduced target strength that can suffice in many practical applications. Ramadan et al.^34^ presented time-harmonic finite-element (FEM) models for 2D and 3D flexible cylindrical and spherical acoustic cloaks based on both approximations and ideal CTs. The influence of structural vibrations on the performance of acoustic cloaks were investigated. Munteanu and Chiroiu^14^ investigated the sound invisibility performance of a 3D spherical machinery cloak comprised of alternating concentric piezoelectric ceramic and epoxy resin layers based on spatial compression with concave-down transformation. Xu et al.^35^ proposed a nonsingular multi-layered cylindrical configuration consisting of two alternately arranged complementary media to reduce scattering from an acoustic sensor while allowing it to receive external information. Jo and Oh^36^ applied impedance and sound speed matching to propose an imperfect multi-layered acoustic shell cloak for a two-dimensional cloaking zone based on feasible material properties and Cummer-Schurig cloaking model. Jo et al.^37^ designed a practically realizable 2D omnidirectional multilayered acoustic shell cloak comprised of axisymmetric cylindrical lattice structures based on a parametric optimization method. The COMSOL multiphysics software in collaboration with a standard optimization technique were employed for obtaining the optimum state of design variables (i.e., layer spacing, cylinder size, and material properties). Zhu, et al.^38^ theoretically studied arbitrary shaped 2D acoustic cloaks operating in a complex inhomogeneous background medium that is comprised of alternating concentric isotropic layers based on CT and transformation acoustic (TA) theory. The superior cloaking performance and feasibility of theoretical design were established via numerical simulations. Li and Vipperman^39^ proposed 2D acoustic cloaks of arbitrary shapes constructed from homogeneous anisotropic parts based on a two-step transformation acoustics method. The cloak was divided into sections, each of which in turn was further divided into two parts, followed by the application of TA theory to derive the required cloaking properties. Two alternating layers of isotropic and homogeneous materials were used to realize the properties of each part of the cloak, while COMSOL Multiphysics finite element software was employed to design and simulate two distinct models in order to illustrate the method. Li et al.^11^ used the effective medium approximation, acoustic scattering theory, and the partial waves series expressions (PWSE) method to design a 3D underwater spherical acoustic cloak comprised of homogeneous isotropic concentric multilayered materials. The cloak was demonstrated to perform very well in concealing the objects under insonification by low frequency acoustic beams. Brisan et al.^9^ used a coordinate concave-down transformation to design a 3D spherical acoustic invisibility cloak with layered metamaterials composed of a triadic Cantor sequence followed by alternating concentric layers of epoxy resin and piezoelectric ceramics. Chen et al.^40^ semi-analytically investigated the effects of material parameters, inner boundary constraints, and damping, on the cloaking performance of a cylindrical acoustic cloak comprised of non-ideal pentamode (PM) materials. It was found that the best broadband cloaking performance occurs with the radially fixed inner constraint, while the PM shear rigidity reduces broadband effectiveness of the cloak by introducting intense low frequency resonances. Nie et al.^41^ theoretically assessed the cloaking performance of an imperfect 3D spherical cloak comprised of non-ideal PMs with small shear rigidity based on the state-space approach. The effects of inner boundary constraints as well as material imperfectness on the cloaking performance were systematically investigated. Furthermore, the critical material imperfectness parameter and the preferable constraint type for broadband invisibility were ascertained. Pomot et al.^42^ combined genetic algorithm (GA) with geometrical transformations to present a general approach for mimicing complex anisotropic inhomogeneous media and applied it to the case of cylindrical acoustic cloaking. Three different types of metamaterial-based acoustic cloaks made of isotropic concentric layers (i.e., split rings, curved rectangles, and crosses) with perforations were designed and studied. Bai et al.^43^ proposed an inclusive CT-based theoretical study on suppression of the radiation characteristics of an underwater acoustic source supplemented by a distantly placed cylindrical acoustic superscatterer that includes an internal core and a double-negative acoustic metamaterial coating. Omnidirectional radiation suppression with coherent extinction was realized by appropriate placement of the acoustic superscatterer while the effectiveness of adopted approach for handling various types of underwater sources (i.e., vibrating rods and ideal monopoles/dipoles) was systematically examined.

Even with the advantage of exotic metamaterials and in spite of its sophisticated mathematical formulation, the CT-based acoustic cloaking faces major challenges to its real-world realization (e.g., extremely high anisotropy, narrowband frequency operation, and unrealistic material parameters), particularly within aqueous surroundings^44^. Consequently, other conceptually different means of passive acoustic concealing techniques such as the SC-based cloaking (also known as plasmonic cloaking) have been suggested. In the SC-based cloaking systems, in contrast to the CT-based cloaking approaches where the interactions of the incident waves with the object is prohibited, only the scattered field in the surrounding medium is eradicated. This way, extreme value parameters may be circumvented based on sophisticated scattering optimization schemes through optimal arrangement of secondary obstacles and/or by optimal tailoring of the neighboring sub-wavelength structure. Numerous researchers have contributed to the subject. For example, García-Chocano et al.^45^ experimentally designed a 2D acoustic cloak operative in a narrow band of incident wave frequencies based on the cancellations from optimally arranged cylinders. Sanchis et al.^23^ experimentally investigated a low loss axisymmetric directional acoustic cloaking design fabricated from 60 concentric acoustically rigid tori that are constructed around a 3D spherical object in air. A GA-based optimization technique was employed to determine the positions and major radii of the tori along the cloak symmetry axis based on complete cancellation of the scattered sound field at a fixed frequency. Up to 90% reduction in the scattering cross section of the sphere was observed at 8.55 kHz. In a series of research works, Guild et al. used SC-based formulation techniques in theoretical design of bilaminate plasmonic-type acoustic cloaks for an arbitrary spherical scatterer^46^, an acoustic sensor represented by a lossy hollow piezoelectric shell^24^, and also for cases of multilayered spherical scatterers, non-spherical objects, and collections of closely packed objects in water^44^. The obtained analytical results were confirmed using 3D FEM simulations. Avital and Miloh^47^ both numerically and theoretically studied effective (near- and far-field) cancellation of sound scattering from a single flexible spherical shell in the vicinity of a free surface under a monochromatic planar incident wave based on optimally configured pressure actuators. The cloaking effectiveness was found to mildly increase as the sphere was placed nearby the free surface. Rohde et al.^48^ presented an experimental demonstration of omnidirectional underwater acoustic scattering cancellation for an array of hollow steel cylinders coated with thin “low shear” elastomeric external layers that are fabricated from silicone rubber. The jointly applied impedance- and sound speed-matched compliant coating resulted in significantly reduced monopole and dipole scattering effects, while the experimentally obtained scattering reductions were confirmed against the FEM modeling predictions. Dutrion and Simon^49^ studied airborne acoustic scattering reduction from a rigid cylinder at a fixed frequency or over a large frequency band by using an optimally-design bi-layer elastic cylindrical shell cloak. Lu et al.^50^ developed an optimization procedure for design, construction, and experimental characterization of a narrowband 2D axisymmetric acoustic SC cloak with a minimum number of Bézier scatterers. More recently, Fujii et al.^51^ used structural topology optimizations based on the covariance matrix adaptation evolution strategy for realizing optimally designed acoustic cloaks that can portray a cylindrical object untraceable in air- and water-borne sound fields. The optimal topologies that minimize the scattering of incident sound waves around acoustic cloaks made from acrylonitrile butadiene styrene copolymers were examined.

The traditional passive acoustic cloaking measures do not require any sophisticated digital signal processing (DSP) equipment and/or external energy consumption which implicate lower complexity and practical implementation costs compared to the semi-active or active methodologies. However, they are not adaptable with respect to changes in the incident sound field and/or scatterer physical characteristics, and often lead to undesirably large scaled designs at low frequencies (long wavelengths). Consequently, the more robust and authoritative active cloaking procedures that can operate based on the conventional or advanced control strategies may be employed to provide a broader cloaking bandwidth at the cost of higher energy expenditure and system complexity^52–54^. Several researchers have recently deliberated on the subject. For example, in a series of research papers, Eggler and coworkers theoretically studied active acoustic cloaking for a rigid cylinder in a convected flow field with circumferentially arranged monopole control sources and error sensors^55^, active optimal noise cloaking of rigid and elastic cylindrical shells with circumferentially arranged external monopole control sources or directly applied structural control forces and discrete error sensors^56^, and active optimal acoustic cloaking and illusions of 3D sound-hard bodies (e.g., rigid spheres and cubes) with spherically arranged external discrete monopole control sources and error sensors^57^. It was demonstrated that overlooking the flow effects can cause amplification of the controlled acoustic field due to the constructive interference between the secondary and primary scattered fields. Also, the applied structural point forces can achieve larger suppression of the scattered sound field than the acoustic monopole control sources typically used in the conventional ANC techniques, particularly when aiming for cloaking of the global exterior domain with reduced control effort. Becker et al.^58^ experimentally rendered broadband active acoustic invisibility and illusion without prior knowledge of the wave field in real time by replacing the physical scattering medium with a virtual homogeneous background medium. The presented results demonstrated effectiveness over a wide frequency range of more than 3.5 octaves as designated with the upper frequency limits of 8.7 kHz (for cloaking) and 5.9 kHz (for holography). Lastly, Hasheminejad and Jamalpoor^54^ investigated active cancellation of acoustic wave scattering from a smart hybrid ERF/PZT double-wall concentric sandwich spherical shell structure based on the approximate thin shell theory and the standard sliding mode control (SMC) scheme. The latter authors obtained acceptable acoustic cloaking performance only within certain narrow band incident wave frequencies.

The preceding brief overview indicates there exist a massive bulk of research studies that adopt a wide assortment of different active or passive cloaking strategies to reduce or completely eradicate incident wave scattering from multilayered structures. Nevertheless, it appears that the three dimensional broadband acoustic cloaking based on a smart hybrid alternate multimorph piezoelectric/viscoelastic (PZT/SVE) actuation configuration has not yet been systematically attempted in any canonical coordinate system. The prime objective of current paper is to investigate this motivating breach in the literature using four dissimilar classes of functional SVE interlayer materials (i.e., MRE, SMP, ERF, and MRSTPF) in a hybrid active/semi-active multi-control framework. Such hybrid multimorph actuation configuration is expected to yield improved cloaking characteristics such as broadband insensitivity to variations in the incident wave direction and frequency, quick response time, large output force/displacement, and reduced operating voltage^59–63^. It can also serve as a practical substitute for the largely narrow-band and invasive scattering cancellation (SC)-based techniques that are highly sensitive to incident wave directionality. Furthermore, the proposed discretely layered two-component cloaking design has cost-effective realizability with available realistic and functional smart materials. It can particularly replace the exotic coordinate transformation (CT)-based acoustic metamaterials that are restrained with extreme material parameters, singularities, high absorption, and narrowband efficiency, especially for the spherically geometry that calls for extremely high inhomogeneity and anisotropy levels which are currently very hard to realize^64^. Lastly, the presented extensive set of numerical simulation data can complement the future experimental results besides serving as an illustrative benchmark for approximate asymptotic and/or strictly numerical methods.

## Mathematical model and method

**Figure 1:**
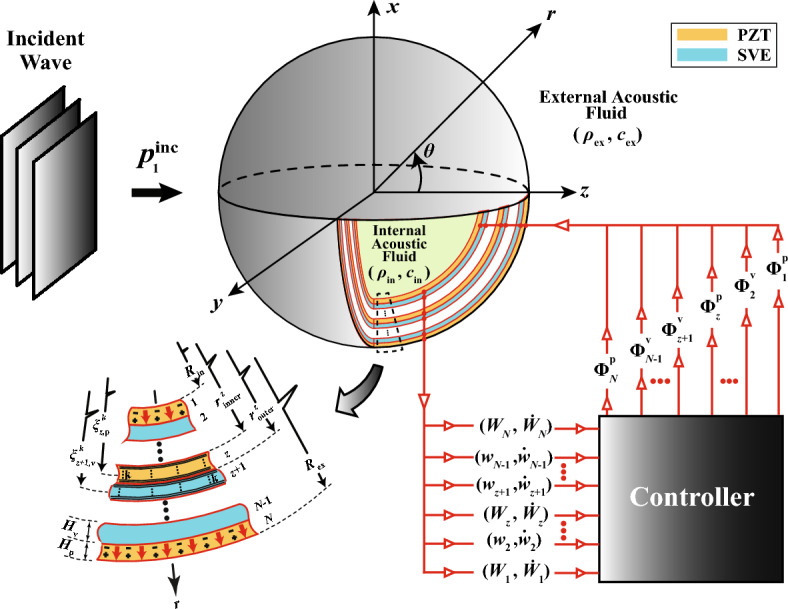
Schematic diagram of the smart hybrid parallel-connected multimorph piezoelectric-viscoelastic spherical cloak structure.

Figure 1 depicts the schematic representation of a nested concentric multimorph hollow sphere of internal radius ${R}_{\text{in}}$ and external radius ${R}_{\text{ex}}$. It is comprised of a strictly periodic sequence of alternate complementary smart piezoelectric/viscoelastic (PZT/SVE) layers of equal thickness ${H}_{\text{p}}={H}_{\text{v}}=H ={(R}_{\text{ex}}$ − ${R}_{\text{in}})/N$, with *N* being the total number of layers, and with the successive layers being labeled with $z=\text{1,2},3,\ldots ,N$ starting from the innermost layer. All of the PZT layers $(z=1, 3,\ldots ,N)$ are assumed to be polarized in the radial direction and electrically connected in the preferred parallel configuration in order to make full use of the actuation voltage^8,59,61,65,66^. Also, the rheological properties of SVE core layers $\left(z=2, 4,\ldots ,N-1\right)$ can be reversibly varied in a controlled fashion based on the externally applied (electric, magnetic, or temperature) field^67^. Furthermore, the thick-walled spherical shell transducer is supposed to be submerged in and filled with ideal compressible acoustic fluids of characteristic impedance $\left({\rho }_{\text{ex}}{c}_{\text{ex}}\right)$ and $\left({\rho }_{\text{in}}{c}_{\text{in}}\right),$ while being insonified along the *z-*direction by a time-harmonic plane incident acoustic wave, ${p}_{1}^{\text{inc}}$. Therefore, based on the standard linear acoustic formulation, the propagation of axially symmetric sound waves (i.e., independent of azimuthal coordinate) in the outer and inner fluid mediums (mediums 1 and 2) are respectively expressed in the forms^68^:

$${c}_{\text{ex}}^{2}\left[\frac{{\partial }^{2}}{\partial {r}^{2}}\left({p}_{1}^{\text{inc}}+{p}_{1}^{\text{scat}}\right)+\frac{2}{r}\frac{\partial }{\partial r}\left({p}_{1}^{\text{inc}}+{p}_{1}^{\text{scat}}\right)+\text{cot}\theta \frac{\partial }{{r}^{2}\partial \theta }\left({p}_{1}^{\text{inc}}+{p}_{1}^{\text{scat}}\right)+\frac{1}{{r}^{2}}\frac{{\partial }^{2}}{\partial {\theta }^{2}}\left({p}_{1}^{\text{inc}}+{p}_{1}^{\text{scat}}\right)\right]=\frac{{\partial }^{2}}{\partial {t}^{2}}\left({p}_{1}^{\text{inc}}+{p}_{1}^{\text{scat}}\right),$$

1 $${c}_{\text{in}}^{2}\left[\frac{{\partial }^{2}}{\partial {r}^{2}}{p}_{2}^{\text{trans}}+\frac{2}{r}\frac{\partial }{\partial r}{p}_{2}^{\text{trans}}+\text{cot}\theta \frac{\partial }{{r}^{2}\partial \theta }{p}_{2}^{\text{trans}}+\frac{1}{{r}^{2}}\frac{{\partial }^{2}}{\partial {\theta }^{2}}{p}_{2}^{\text{trans}}\right]=\frac{{\partial }^{2}}{\partial {t}^{2}}{p}_{2}^{\text{trans}},$$

where $\left({p}_{1}^{\text{inc}},{p}_{1}^{\text{scat}},{p}_{2}^{\text{trans}}\right),$ which refer to the incident, scattered, and transmitted spherical wave, respectively, could profitably be expressed as functions of their modal coordinates in the forms^7,69^:

$${p}_{1}^{\text{inc}}\left(r,\theta ,t\right)={p}_{0}\sum_{n=0}^{\infty }\left(2n+1\right){\text{i}}^{n}{j}_{n}\left({k}_{\text{ex}}r\right){P}_{n}\left(\text{cos}\theta \right){e}^{\text{i}\omega t},$$

$${p}_{1}^{\text{scat}}\left(r,\theta ,t\right)=\sum_{n=0}^{\infty }\left(2n+1\right){\text{i}}^{n}{p}_{1n}^{\text{scat}}{h}_{n}\left({k}_{\text{ex}}r\right){P}_{n}(\text{cos}\theta ){e}^{\text{i}\omega t},$$

2 $${p}_{2}^{\text{trans}}\left(r,\theta ,t\right)=\sum_{n=0}^{\infty }\left(2n+1\right){\text{i}}^{n}{p}_{2n}^{\text{trans}}{j}_{n}\left({k}_{\text{in}}r\right){P}_{n}(\text{cos}\theta ){e}^{\text{i}\omega t},$$

Where $\text{i}=\sqrt{-1}, {p}_{0}$ is the amplitude of incident pressure wave,${P}_{n}$ is the *n-*th order Legendre polynomial^70^, $({ j}_{n},{h}_{n})$ signify the spherical Bessel and Hankel functions of the first kind, respectively, ${k}_{i}=\omega /{c}_{i} (i=\text{ex},\text{in})$ is the acoustic wave number, and $\omega$ is the circular frequency.

### Multimorph piezo-viscoelastic shell

In this subsection, the main equations governing the dynamics of the multi-layered hybrid PZT/SVE hollow sphere will be derived using the exact theory of 3D elasticity. Hence, the basic stress–strain and electric displacement–strain equations associated with the piezo-actuator layers are written in the spherical coordinates as^71^:

3 $$\left[\begin{array}{c}{\Sigma }_{\theta \theta }\\ {\Sigma }_{\phi \phi }\\ {\Sigma }_{rr}\\ {\Sigma }_{r\theta }\\ {\Sigma }_{r\phi }\\ {\Sigma }_{\theta \phi }\end{array}\right]=\left[\begin{array}{cccccc}{C}_{11}& {C}_{12}& {C}_{13}& 0& 0& 0\\ {C}_{12}& {C}_{11}& {C}_{13}& 0& 0& 0\\ {C}_{13}& {C}_{13}& {C}_{33}& 0& 0& 0\\ 0& 0& 0& 2{C}_{44}& 0& 0\\ 0& 0& 0& 0& 2{C}_{44}& 0\\ 0& 0& 0& 0& 0& 2{C}_{66}\end{array}\right]\left[\begin{array}{c}{\text{s}}_{\theta \theta }\\ {\text{s}}_{\phi \phi }\\ {\text{s}}_{rr}\\ {\text{s}}_{r\theta }\\ {\text{s}}_{r\phi }\\ {\text{s}}_{\theta \phi }\end{array}\right]+\left[\begin{array}{c}{e}_{31}{\nabla }_{2}\\ {e}_{31}{\nabla }_{2}\\ {e}_{33}{\nabla }_{2}\\ {e}_{15}\frac{\partial }{\partial \theta }\\ \frac{{e}_{15}}{\text{sin}\theta }\frac{\partial }{\partial \phi }\\ 0\end{array}\right]\Phi$$

$$\left[\begin{array}{c}{D}_{\theta }\\ {D}_{\phi }\\ {D}_{r}\end{array}\right]=\frac{1}{r}\left[\begin{array}{c}0\\ 0\\ {e}_{31}\end{array}\begin{array}{c}0\\ 0\\ {e}_{31}\end{array}\begin{array}{c}0\\ 0\\ {e}_{33}\end{array}\begin{array}{c}{2e}_{15}\\ 0\\ 0\end{array}\begin{array}{c}0\\ {2e}_{15}\\ 0\end{array}\begin{array}{c}0\\ 0\\ 0\end{array}\right]\left[\begin{array}{c}{\text{s}}_{\theta \theta }\\ {\text{s}}_{\phi \phi }\\ {\text{s}}_{rr}\\ {\text{s}}_{r\theta }\\ {\text{s}}_{r\phi }\\ {\text{s}}_{\theta \phi }\end{array}\right]-\frac{1}{r}\left[\begin{array}{c}{\varepsilon }_{11}\frac{\partial }{\partial \theta }\\ \frac{{\varepsilon }_{11}}{\text{sin}\theta }\frac{\partial }{\partial \theta }\\ {\varepsilon }_{11}{\nabla }_{2}\end{array}\right]\Phi ,$$

where $\Phi$ is the electric potential, ${\nabla }_{2}=r\frac{\partial }{\partial r}$, $2{C}_{66}={C}_{11}-{C}_{12},$ $(\text{S},\Sigma ,D)$ signify the strain, stress, and electric displacement, while ${C}_{ij}$,${e}_{ij}$ and ${\varepsilon }_{ij}$ refer to the elastic, piezoelectric, and dielectric constants, respectively. Also, the pertinent strain–displacement is written as

4 $$\left[\begin{array}{c}{\text{S}}_{\theta \theta }\\ {\text{s}}_{\phi \phi }\\ {\text{s}}_{rr}\\ {\text{s}}_{r\theta }\\ {\text{s}}_{r\phi }\\ {\text{s}}_{\theta \phi }\end{array}\right]=\left[\begin{array}{ccc}\frac{\partial }{\partial \theta }& 0& 1\\ \text{cot}\theta & \frac{1}{\text{sin}\theta }\frac{\partial }{\partial \phi }& 1\\ 0& 0& {\nabla }_{2}\\ {\nabla }_{2}-1& 0& \frac{\partial }{\partial \theta }\\ 0& {\nabla }_{2}-1& \frac{1}{\text{sin}\theta }\frac{\partial }{\partial \phi }\\ \frac{1}{\text{sin}\theta }\frac{\partial }{\partial \phi }& \frac{\partial }{\partial \theta }-\text{cot}\theta & 0\end{array}\right]\left[\begin{array}{c}{U}_{\theta }\\ {U}_{\phi }\\ {U}_{r}\end{array}\right],$$

where $U$ signify piezo-actuator displacement. In addition, assuming no body forces, the core governing response equations are given as^71^:

$$\frac{\partial {\Sigma }_{\theta \theta }}{\partial \theta }+{\Sigma }_{\theta \theta }\text{cot}\theta -{\Sigma }_{\phi \phi }\text{cot}\theta +\left({\nabla }_{2}+2\right){\Sigma }_{r\theta }+\frac{\partial {\Sigma }_{\theta \phi }}{\partial \phi }\text{csc}\theta ={\rho }_{\text{p}}{r}^{2}\frac{{\partial }^{2}{U}_{\theta }}{\partial {t}^{2}} ,$$

$$\frac{\partial {\Sigma }_{\phi \phi }}{\partial \phi }\text{csc}\theta +2{\Sigma }_{r\theta }+{\nabla }_{2}{\Sigma }_{r\theta }+\frac{\partial {\Sigma }_{\theta \phi }}{\partial \theta }+2{\Sigma }_{\theta \phi }\text{cot}\theta ={\rho }_{\text{p}}{r}^{2}\frac{{\partial }^{2}{U}_{\phi }}{\partial {t}^{2}} ,$$

5 $$-{\Sigma }_{\theta \theta }-{\Sigma }_{\phi \phi }+\left({\nabla }_{2}+1\right){\Sigma }_{rr}+\frac{\partial {\Sigma }_{r\theta }}{\partial \theta }+{\Sigma }_{{r }\theta}\text{cot}\theta +\frac{\partial {\Sigma }_{r\phi }}{\partial \phi }\text{csc}\theta ={\rho }_{\text{p}}{r}^{2}\frac{{\partial }^{2}{U}_{r}}{\partial {t}^{2}} ,$$

where ${\rho }_{\text{p}}$ is mass density of piezoelectric layer, and the electric equilibrium equation in the absence of free charge density is written as

6 $$r\frac{\partial }{\partial r}{D}_{r}+{D}_{r}+\frac{1}{\text{sin}\theta }\frac{\partial }{\partial \theta }\left({D}_{\theta }\text{sin}\theta \right)+\frac{1}{\text{sin}\theta }\frac{\partial {D}_{\phi }}{\partial \phi }=0.$$

Next, the stress–strain constitutive relationship for the homogeneous isotropic SVE layers can be presented in the standard matrix form^72^:

7 $$\left[\begin{array}{c}{\upsigma }_{\theta \theta }\\ {\upsigma }_{\phi \phi }\\ {\upsigma }_{rr}\\ {\upsigma }_{r\theta }\\ {\upsigma }_{r\phi }\\ {\upsigma }_{\theta \phi }\end{array}\right]=\left[\begin{array}{cccccc}{\lambda }_{\text{v}}^{*}+2{\mu }_{\text{v}}^{*}& {\lambda }_{\text{v}}^{*}& {\lambda }_{\text{v}}^{*}& 0& 0& 0\\ {\lambda }_{\text{v}}^{*}& {\lambda }_{\text{v}}^{*}+2{\mu }_{\text{v}}^{*}& {\lambda }_{\text{v}}^{*}& 0& 0& 0\\ {\lambda }_{\text{v}}^{*}& {\lambda }_{\text{v}}^{*}& {\lambda }_{\text{v}}^{*}+2{\mu }_{\text{v}}^{*}& 0& 0& 0\\ 0& 0& 0& 2{\mu }_{\text{v}}^{*}& 0& 0\\ 0& 0& 0& 0& 2{\mu }_{\text{v}}^{*}& 0\\ 0& 0& 0& 0& 0& 2{\mu }_{\text{v}}^{*}\end{array}\right]\left[\begin{array}{c}{\text{s}}_{\theta \theta }\\ {\text{s}}_{\phi \phi }\\ {\text{s}}_{rr}\\ {\text{s}}_{r\theta }\\ {\text{s}}_{r\phi }\\ {\text{s}}_{\theta \phi }\end{array}\right],$$

where ($\text{s},\sigma )$ refer to the strain and stress in the SVE layers, respectively, $({\lambda }_{\text{v}}^{*}, {\mu }_{\text{v}}^{*}$) = $\left(\frac{2{\upsilon }_{\text{v}}}{1-2{\upsilon }_{\text{v}}} {G}_{\text{v}}^{*} , {G}_{\text{v}}^{*}\right)=\left( \frac{{\upsilon }_{\text{v}}}{(1+{\upsilon }_{\text{v}})(1-2{\upsilon }_{\text{v}})} {E}_{\text{v}}^{*} , {\frac{1}{2(1+{\upsilon }_{\text{v}})}E}_{\text{v}}^{*}\right)$ signify the complex Lame constants, ${\upsilon }_{\text{v}}$ is the Poisson ratio, and ${(G}_{\text{v}}^{*},$ ${E}_{\text{v}}^{*})$ refer to the complex moduli of the SVE which will be defined later. Also, the pertinent matrix strain–displacement relation is given as

8 $$\left[\begin{array}{c}{\text{s}}_{\theta \theta }\\ {\text{s}}_{\phi \phi }\\ {\text{s}}_{rr}\\ {\text{s}}_{r\theta }\\ {\text{s}}_{r\phi }\\ {\text{s}}_{\theta \phi }\end{array}\right]=\left[\begin{array}{ccc}\frac{\partial }{\partial \theta }& 0& 1\\ \text{cot}\theta & \frac{1}{\text{sin}\theta }\frac{\partial }{\partial \phi }& 1\\ 0& 0& {\nabla }_{2}\\ {\nabla }_{2}-1& 0& \frac{\partial }{\partial \theta }\\ 0& {\nabla }_{2}-1& \frac{1}{\text{sin}\theta }\frac{\partial }{\partial \phi }\\ \frac{1}{\text{sin}\theta }\frac{\partial }{\partial \phi }& \frac{\partial }{\partial \theta }-\text{cot}\theta & 0\end{array}\right]\left[\begin{array}{c}{u}_{\theta }\\ {u}_{\phi }\\ {u}_{r}\end{array}\right],$$

where $u$ is displacement. Furthermore, with lack of body forces, the governing dynamic response equations for SVE layers are written as^71^:

$$\frac{\partial {\upsigma }_{\theta \theta }}{\partial \theta }+{\upsigma }_{\theta \theta }\text{cot}\theta -{\upsigma }_{\phi \phi }\text{cot}\theta +\left({\nabla }_{2}+2\right){\upsigma }_{r\theta }+\frac{\partial {\upsigma }_{\theta \phi }}{\partial \phi }\text{csc}\theta ={\rho }_{\text{v}}{r}^{2}\frac{{\partial }^{2}{u}_{\theta }}{\partial {t}^{2}} ,$$

$$\frac{\partial {\upsigma }_{\phi \phi }}{\partial \phi }\text{csc}\theta +2{\upsigma }_{r\theta }+{\nabla }_{2}{\upsigma }_{r\theta }+\frac{\partial {\upsigma }_{\theta \phi }}{\partial \theta }+2{\upsigma }_{\theta \phi }\text{cot}\theta ={\rho }_{\text{v}}{r}^{2}\frac{{\partial }^{2}{u}_{\phi }}{\partial {t}^{2}} ,$$

9 $$-{\upsigma }_{\theta \theta }-{\upsigma }_{\phi \phi }+\left({\nabla }_{2}+1\right){\upsigma }_{rr}+\frac{\partial {\upsigma }_{r\theta }}{\partial \theta }+{\upsigma }_{{r }\theta}\text{cot}\theta +\frac{\partial {\upsigma }_{r\phi }}{\partial \phi }\text{csc}\theta ={\rho }_{\text{v}}{r}^{2}\frac{{\partial }^{2}{u}_{r}}{\partial {t}^{2}} ,$$

where ${\rho }_{\text{v}}$ refer to the mass density of the SVE layers.

Next, it is advantageous to introduced a new group of stress and displacement field potentials $(\psi , g, w, \Psi , G,W; {\upsigma }_{1}, {\upsigma }_{2}, {\Sigma }_{1}$,${\Sigma }_{2})$ as^73^:

10 $$\begin{aligned}& {\Sigma }_{r\theta }=-\frac{1}{\text{sin}\theta }\frac{\partial {\Sigma }_{1}}{\partial \phi }-\frac{\partial {\Sigma }_{2}}{\partial \theta }, \quad \quad {\upsigma }_{r\theta }=-\frac{1}{\text{sin}\theta }\frac{\partial {\upsigma }_{1}}{\partial \phi }-\frac{\partial {\upsigma }_{2}}{\partial \theta },\\ &{\Sigma }_{r\phi }=\frac{\partial {\Sigma }_{1}}{\partial \theta }-\frac{1}{\text{sin}\theta }\frac{\partial {\Sigma }_{2}}{\partial \phi }, { U}_{\phi }=\frac{\partial \Psi }{\partial \theta }-\frac{1}{\text{sin}\theta }\frac{\partial G}{\partial \phi }, \quad \quad {\upsigma }_{r\phi }=\frac{\partial {\upsigma }_{1}}{\partial \theta }-\frac{1}{\text{sin}\theta }\frac{\partial {\upsigma }_{2}}{\partial \phi }, { u}_{\phi }=\frac{\partial \psi }{\partial \theta }-\frac{1}{\text{sin}\theta }\frac{\partial g}{\partial \phi },\\ & {U}_{\theta }=-\frac{1}{\text{sin}\theta }\frac{\partial \Psi }{\partial \phi }-\frac{\partial G}{\partial \theta }, \quad \quad {U}_{r}=W,{u}_{\theta }=-\frac{1}{\text{sin}\theta }\frac{\partial \psi }{\partial \phi }-\frac{\partial g}{\partial \theta }, {u}_{r}=w.\end{aligned}$$

with the following modal expansions for the current axisymmetric configuration (see Fig. 1):

11 $$\begin{aligned}& {\Sigma }_{1}={R}_{\text{in}}{C}_{44}^{\text{in}}\sum_{n=0}^{\infty } {\Sigma }_{1}^{n}\left(\xi \right){P}_{n}\left(\text{cos}\theta \right), \quad \Phi ={R}_{\text{in}}\frac{{e}_{33}^{\text{in}}}{{\varepsilon }_{33}^{\text{in}}}\sum_{n=0}^{\infty } {\Phi }^{n}\left(\xi \right){P}_{n}\left(\text{cos}\theta \right),\\ & \Psi ={R}_{\text{in}}\sum_{n=0}^{\infty } {\Psi }^{n}\left(\xi \right){P}_{n}\left(\text{cos}\theta \right), \quad {\upsigma }_{1}={R}_{\text{in}}{C}_{44}^{\text{in}}\sum_{n=0}^{\infty } {\upsigma }_{1}^{n}\left(\xi \right){P}_{n}\left(\text{cos}\theta \right),\\ &{\Sigma }_{rr}={R}_{\text{in}}{C}_{44}^{\text{in}}\sum_{n=0}^{\infty } {\Sigma }_{rr}^{n}\left(\xi \right){P}_{n}\left(\text{cos}\theta \right), \quad \psi ={R}_{\text{in}}\sum_{n=0}^{\infty } {\psi }^{n}\left(\xi \right){P}_{n}\left(\text{cos}\theta \right),\\ & {\Sigma }_{2}={R}_{\text{in}}{C}_{44}^{\text{in}}\sum_{n=0}^{\infty } {\Sigma }_{2}^{n}\left(\xi \right){P}_{n}\left(\text{cos}\theta \right), \quad {\upsigma }_{rr}={R}_{\text{in}}{C}_{44}^{\text{in}}\sum_{n=0}^{\infty } {\upsigma }_{rr}^{n}\left(\xi \right){P}_{n}\left(\text{cos}\theta \right),\\ &G={R}_{\text{in}}\sum_{n=0}^{\infty } {G}^{n}\left(\xi \right){P}_{n}\left(\text{cos}\theta \right), \quad {\upsigma }_{2}={R}_{\text{in}}{C}_{44}^{\text{in}}\sum_{n=0}^{\infty } {\upsigma }_{2}^{n}\left(\xi \right){P}_{n}\left(\text{cos}\theta \right),\\ & W={R}_{\text{in}}\sum_{n=0}^{\infty } {W}^{n}\left(\xi \right){P}_{n}\left(\text{cos}\theta \right), \quad g={R}_{\text{in}}\sum_{n=0}^{\infty } {g}^{n}\left(\xi \right){P}_{n}\left(\text{cos}\theta \right),\\ & {D}_{r}={R}_{\text{in}}{e}_{33}^{\text{in}}\sum_{n=0}^{\infty } {D}_{r}^{n}\left(\xi \right){P}_{n}\left(\text{cos}\theta \right), \quad w={R}_{\text{in}}\sum_{n=0}^{\infty } {w}^{n}\left(\xi \right){P}_{n}\left(\text{cos}\theta \right), \end{aligned}$$

where $\xi$ is the dimensionless radial coordinate, and ${(C}_{44}^{\text{in}}, {e}_{33}^{\text{in}}, {\varepsilon }_{33}^{\text{in}})$ are piezo-elastic normalization constants for the inner-most piezoelectric actuator layer (“layer 1”). Subsequent application of the transformation Eq. (10) in conjunction with expansions (Eq. 11) into the governing Eqs. (5) and (9), after some operations, one obtains the separated state-space equations in dimensionless forms^71^:

12 $$\frac{d}{d\xi }{\mathbf{V}}_{1,n}\left(\xi \right)={\mathbf{M}}_{1,n}\left(\xi \right){\mathbf{V}}_{1,n}\left(\xi \right), \quad \frac{d}{d\xi }{\mathbf{V}}_{2,n}\left(\xi \right)={\mathbf{M}}_{2,n}\left(\xi \right) {\mathbf{V}}_{2,n}\left(\xi \right),$$

13 $$\frac{d}{d\xi }{\mathbf{v}}_{1,n}\left(\xi \right)={\mathbf{m}}_{1,n}\left(\xi \right) {\mathbf{v}}_{1,n}\left(\xi \right), \quad \frac{d}{d\xi }{\mathbf{v}}_{2,n}\left(\xi \right)={\mathbf{m}}_{2,n}\left(\xi \right) {\mathbf{v}}_{2,n}\left(\xi \right),$$

where $n=\text{0,1},\text{2,3},\ldots$, the non-zero elements of modal coefficient matrices (${\mathbf{m}}_{1,n}$, ${\mathbf{m}}_{2,n}$, ${\mathbf{M}}_{1,n}$, ${\mathbf{M}}_{2,n})$ are given in Supplementary Material A, and the modal state vectors are defined as

$${\mathbf{V}}_{1,n}={\left[\begin{array}{cc}{\Sigma }_{1}^{n}& {\Psi }^{n}\end{array}\right]}^{T},{\mathbf{V}}_{2,n}={\left[\begin{array}{cccccc}{\Sigma }_{rr}^{n}& {\Sigma }_{2}^{n}& {G}^{n}& {W}^{n}& {D}_{r}^{n}& {\Phi }^{n}\end{array}\right]}^{T},$$

$${\mathbf{v}}_{1,n}={\left[\begin{array}{cc}{\upsigma }_{1}^{n}& {\psi }^{n}\end{array}\right]}^{T},{\mathbf{v}}_{2,n}={\left[\begin{array}{cccc}{\sigma }_{rr}^{n}& {\sigma }_{2}^{n}& {g}^{n}& {w}^{n}\end{array}\right]}^{T}.$$

Now, adopting the classical approximate lamination approach^71^, the piezoelectric and viscoelastic layers are considered to be comprised of ${q}_{\text{P}}^{z}$ and ${q}_{\text{v}}^{z}$ flawlessly joined sublayers with their respective thicknesses defined as

14 $$\begin{aligned}& {\widehat{H}}_{\text{p}}^{z}=({r}_{\text{outer}}^{z}-{r}_{\text{inner}}^{z})/{q}_{\text{p}}^{z}, \quad \left(z=\text{1,3},5,\ldots ,N\right),\\ &{\widehat{H}}_{\text{v}}^{z}=({r}_{\text{outer}}^{z}-{r}_{\text{inner}}^{z})/{q}_{\text{v}}^{z}, \quad (z=\text{2,4},6,\ldots ,N-1), \end{aligned}$$

where ${r}_{\text{outer}}^{z}={r}_{\text{inner}}^{z}+z H (z=\text{1,2},\ldots ,N)$, and one can clearly see from Fig. 1 that ${r}_{\text{inner}}^{1}={R}_{\text{in}},$ ${r}_{\text{outer}}^{z}={r}_{\text{inner}}^{z+1}$, and ${r}_{\text{outer}}^{N}={R}_{\text{ex}}.$ As the thickness of each sub-layer may become very small with increase of $({q}_{\text{P}}^{z},{q}_{\text{v}}^{z})$, the modal coefficient matrices $({\mathbf{m}}_{1,n}$, ${\mathbf{m}}_{2,n}$, ${\mathbf{M}}_{1,n}$, ${\mathbf{M}}_{2,n})$ can be favorably considered invariable within each sublayer. Besides, the nondimensional radius of each sublayer within the viscoelastic and piezoelectric material layers are correspondingly defined as

15 $$\begin{aligned} & {\xi }_{z,\text{p}}^{k}=\text{ln}\frac{{r}_{\text{inner}}^{z}+k{\widehat{H}}_{\text{p}}^{z}}{{r}_{\text{inner}}^{z}+(k-1){\widehat{H}}_{\text{p}}^{z}} , {(\text{k}\hspace{0.17em}=\hspace{0.17em}\text{1,2},\ldots , q}_{\text{p}}^{\text{z}}; \quad z=\text{1,3},\ldots ,N),\\ & {\xi }_{z,\text{v}}^{k}=\text{ln}\frac{{r}_{\text{inner}}^{z}+k{\widehat{H}}_{\text{v}}^{z}}{{r}_{\text{inner}}^{z}+\left(k-1\right){\widehat{H}}_{\text{v}}^{z}},(k =\text{1,2},\ldots , {q}_{\text{v}}^{\text{z}};\quad z=\text{2,4},\ldots ,N-1),\end{aligned}$$

Next, by imposing the interface boundary constraints among all sublayers, the state parameters at the outer surface of each viscoelastic or piezoelectric layer (i.e. at *r* = ${r}_{\text{outer}}^{\text{z}}$, $z=\text{1,2},3,\ldots ,N$) can respectively be linked to those at the inner surface (i.e. at *r* $={r}_{\text{inner}}^{\text{z}}$, $z=\text{1,2},3,\ldots ,N$) in terms of their associated modal (local) transfer matrices in the forms^71^:

$$\begin{aligned}{\mathbf{V}}_{1,n}\left(\xi ={\xi }_{\text{z},\text{p}}^{{q}_{\text{p}}^{\text{z}}}\right)={\bf {\mathcal T}}_{1,n}^{\text{z},\text{p}}{\mathbf{V}}_{1,n}\left(\xi ={\xi }_{\text{z}-1,\text{p}}^{{q}_{\text{p}}^{\text{z}-1}}\right), & {\mathbf{V}}_{\text{2,0}}\left(\xi ={\xi }_{\text{z},\text{p}}^{{q}_{\text{p}}^{\text{z}}}\right)={\bf {\mathcal T}}_{\text{2,0}}^{\text{z},\text{p}}{\mathbf{V}}_{\text{2,0}}\left(\xi ={\xi }_{\text{z}-1,\text{p}}^{{q}_{\text{p}}^{\text{z}-1}}\right),\left(z=\text{1,3},\ldots ,N\right),\\ & {\mathbf{V}}_{2,n}\left(\xi ={\xi }_{\text{z},\text{p}}^{{q}_{\text{p}}^{\text{z}}}\right)={\bf {\mathcal T}}_{2,n}^{\text{z},\text{p}}{\mathbf{V}}_{2,n}\left(\xi ={\xi }_{\text{z}-1,\text{p}}^{{q}_{\text{p}}^{\text{z}-1}}\right), \left(z=\text{1,3},\ldots ,N\right),\end{aligned}$$

16 $$\begin{aligned} {\mathbf{v}}_{1,n}\left(\xi ={\xi }_{\text{z},\text{v}}^{{q}_{\text{v}}^{\text{z}}}\right)={\bf{\mathcalligra t}}_{1,n}^{\text{z},\text{v}} {\mathbf{v}}_{1,n}\left(\xi ={\xi }_{\text{z}-1,\text{v}}^{{q}_{\text{v}}^{\text{z}-1}}\right),&{\mathbf{v}}_{\text{2,0}}\left(\xi ={\xi }_{\text{z},\text{v}}^{{q}_{\text{v}}^{\text{z}}}\right)={\bf{\mathcalligra t}}_{\text{2,0}}^{\text{z},\text{v}} {\mathbf{v}}_{\text{2,0}}\left(\xi ={\xi }_{\text{z}-1,\text{v}}^{{q}_{\text{v}}^{\text{z}-1}}\right),\left( z=\text{2,4},\ldots ,N-1\right), \\ & {\mathbf{v}}_{2,n}\left(\xi ={\xi }_{\text{z},\text{v}}^{{q}_{\text{v}}^{\text{z}}}\right)={\bf{\mathcalligra t}}_{2,n}^{\text{z},\text{v}} {\mathbf{v}}_{2,n}\left(\xi ={\xi }_{\text{z}-1,\text{v}}^{{q}_{\text{v}}^{\text{z}-1}}\right),\left( z=\text{2,4},\ldots ,N-1\right),\end{aligned}$$

where,$${\xi }_{0,\text{p}}^{{q}_{\text{p}}^{0}}={\xi }_{1,\text{p}}^{1}=\text{ln}\frac{{R}_{\text{in}}+{\widehat{H}}_{\text{p}}^{\text{z}}}{{R}_{\text{in}}}$$, $${\mathbf{V}}_{\text{2,0}}={\left[\begin{array}{cccc}{\Sigma }_{rr}^{0}& {W}^{0}& {D}_{r}^{0}& {\Phi }^{0}\end{array}\right]}^{T}$$, $${\mathbf{v}}_{\text{2,0}}={\left[\begin{array}{cc}{\sigma }_{rr}^{0}& {w}^{0}\end{array}\right]}^{T}$$, and

$${\bf {\mathcal T}}_{1,n}^{\text{z},\text{p}}={\prod }_{k={q}_{\text{p}}^{\text{z}}}^{1}\text{exp}[{\xi }_{\text{z},\text{p}}^{\text{k}}{\mathbf{M}}_{1,n}\left({\xi }_{\text{z},\text{p}}^{\text{k}}\right)],{\bf{\mathcalligra t}}_{1,n}^{\text{z},\text{v}}={\prod }_{k={q}_{\text{v}}^{\text{z}}}^{1}\text{exp}\left[{\xi }_{\text{z},\text{v}}^{\text{k}}{ \mathbf{m}}_{1,n}\left({\xi }_{\text{z},\text{v}}^{\text{k}}\right)\right],$$

$${\bf {\mathcal T}}_{\text{2,0}}^{\text{z},\text{p}}={\prod }_{k={q}_{\text{p}}^{\text{z}}}^{1}\text{exp}[{\xi }_{\text{z},\text{p}}^{\text{k}}{\mathbf{M}}_{\text{2,0}}\left({\xi }_{\text{z},\text{p}}^{\text{k}}\right)],{\bf{\mathcalligra t}}_{\text{2,0}}^{\text{z},\text{v}}={\prod }_{k={q}_{\text{v}}^{\text{z}}}^{1}\text{exp}[{\xi }_{\text{z},\text{v}}^{\text{k}}{ \mathbf{m}}_{\text{2,0}}\left({\xi }_{\text{z},\text{v}}^{\text{k}}\right)],$$

$${\bf {\mathcal T}}_{2,n}^{\text{z},\text{p}}={\prod }_{k={q}_{\text{p}}^{\text{z}}}^{1}\text{exp}[{\xi }_{\text{z},\text{p}}^{\text{k}}{\mathbf{M}}_{2,n}\left({\xi }_{\text{z},\text{p}}^{\text{k}}\right)],{\bf{\mathcalligra t}}_{2,n}^{\text{z},\text{v}}={\prod }_{k={q}_{\text{v}}^{\text{z}}}^{1}\text{exp}\left[{\xi }_{\text{z},\text{v}}^{\text{k}}{ \mathbf{m}}_{2,n}\left({\xi }_{\text{z},\text{v}}^{\text{k}}\right)\right],$$

in which $n=\text{1,2},3,\ldots$, and as with (${\mathbf{m}}_{1,n}$, ${\mathbf{m}}_{2,n}$, ${\mathbf{M}}_{1,n}$, ${\mathbf{M}}_{2,n}),$ the non-zero elements of the remaining modal coefficient matrices $({\mathbf{M}}_{\text{2,0}},{\mathbf{m}}_{\text{2,0}})$ are given in Supplementary Material A.

In case of heavy fluid loading (e.g., when the piezo-laminated shell is submerged in water), the structural and fluid motion get coupled at the structure-fluid interface in a complicated manner. This will result in the fluid coupled system matrices of the hybrid multi-layered structure to become complex valued owing to the fluid loading effects and decay of energy linked to the external pressure radiation. In particular, for an ideal non-viscous acoustic fluid, the fluid-loaded structural modes are known to expediently decouple into two independent classes of vibrations, namely, the first-class (torsional or toroidal) modes that are associated to equi-volumetric motion, and the second-class (spheroidal) modes that are directly impelled by the fluid loading. This way, as it has also been demonstrated in Refs.,^74,75^ only the second class modes of structural vibrations associated with (${\mathbf{V}}_{2,n},{\mathbf{v}}_{2,n};n=\text{0,1},\text{2,3},\ldots$) can get excited in the present ideally coupled FSI problem. Hence, after imposing the required global PZT/SVE interface compatibility conditions, the state space solutions (16), with some significant manipulations, reduce to:

$${\overline{\mathbf{V}} }_{\text{2,0}}\left(\xi ={\xi }_{\text{N},\text{p}}^{{q}_{\text{p}}^{\text{N}}}\right)={\bf {\mathcal G}}_{0}{\overline{\mathbf{V}} }_{\text{2,0}}\left(\xi ={\xi }_{1,\text{p}}^{1}\right)+\sum_{z=1}^{N}{\bf {\mathcal B}}_{\text{z}}^{0},$$

17 $${\overline{\mathbf{V}} }_{2,n}\left(\xi ={\xi }_{\text{N},\text{p}}^{{q}_{\text{p}}^{\text{N}}}\right)={\bf {\mathcal G}}_{n}{\overline{\mathbf{V}} }_{2,n}\left(\xi ={\xi }_{1,\text{p}}^{1}\right)+\sum_{z=1}^{N}{\bf {\mathcal B}}_{\text{z}}^{n}, \left(\text{z}=\text{1,3},\ldots , N\right),$$

where $${\overline{\mathbf{V}} }_{\text{2,0}}={\left[\begin{array}{cc}{\Sigma }_{rr}^{0}& {W}^{0}\end{array}\right]}^{T}$$ and $${\overline{\mathbf{V}} }_{2,n}={\left[\begin{array}{cccc}{\Sigma }_{rr}^{n}& {\Sigma }_{2}^{n}& {G}^{n}& {W}^{n}\end{array}\right]}^{T}$$ stand for the mechanical share of state vectors (${\mathbf{V}}_{\text{2,0}},{\mathbf{V}}_{2,n})$, respectively, and the associated global transfer matrix, ${\bf {\mathcal G}}_{n},$ is given as

18 $${\bf {\mathcal G}}_{n}={\overline{\bf {\mathcal T}} }_{2,n}^{ N,\text{p}}{\bf{\mathcalligra t}}_{2,n}^{ N-1,\text{v}}{ \overline{\bf {\mathcal T}} }_{2,n}^{ N-2,\text{p}} {\bf{\mathcalligra t}}_{2,n}^{ N-3,\text{v}}\ldots {\bf{\mathcalligra t}}_{2,n}^{ N-\left(\text{N}-2\right),\text{v}} {\overline{\bf {\mathcal T}} }_{2,n}^{ N-\left(N-1\right),\text{p}},$$

in which $n=\text{0,1},2,\ldots ,$ and

$${\overline{\bf {\mathcal T}} }_{\text{2,0}}^{\text{ z},\text{p}}={\bf {\mathcal T}}_{\text{2,0}}^{\text{z},\text{p}}\left(1:\text{2,1}:2\right)-{\left[{\bf {\mathcal T}}_{\text{2,0}}^{\text{z},\text{p}}\left(\text{4,3}\right)\right]}^{-1} {\bf {\mathcal T}}_{\text{2,0}}^{\text{z},\text{p}}\left(1:\text{2,3}\right) {\bf {\mathcal T}}_{\text{2,0}}^{\text{z},\text{p}}\left(\text{4,1}:2\right),$$

19 $${\overline{\bf {\mathcal T}} }_{2,n}^{\text{ z},\text{p}}={\bf {\mathcal T}}_{2,n}^{\text{z},\text{p}}\left(1:\text{4,1}:4\right)-{\left[{\bf {\mathcal T}}_{2,n}^{\text{z},\text{p}}\left(\text{6,5}\right)\right]}^{-1} {\bf {\mathcal T}}_{2,n}^{\text{z},\text{p}}\left(1:\text{4,5}\right) {\bf {\mathcal T}}_{2,n}^{\text{z},\text{p}}\left(\text{6,1}:4\right).(\text{z}=\text{1,3},\ldots , N),$$

Furthermore, after adopting the efficient parallel connection actuator configuration for the present multimorph piezoelectric cloak structure^8,76–78^, after some manipulations, the residual matrix coefficients $({\bf {\mathcal B}}_{\text{z}}^{0},{\bf {\mathcal B}}_{\text{z}}^{n})$ are obtained as

$${\bf {\mathcal B}}_{\text{z}}^{0}=\frac{{\Phi }_{\text{z}}^{0,\text{p}}}{H}\left\{{\bf {\mathcal T}}_{\text{2,0}}^{\text{z},\text{p}}\left(1:\text{2,4}\right)-\left[{\bf {\mathcal T}}_{\text{2,0}}^{\text{z},\text{p}}\left(\text{4,4}\right)/{\bf {\mathcal T}}_{\text{2,0}}^{\text{z},\text{p}}\left(\text{4,3}\right)\right]{\bf {\mathcal T}}_{\text{2,0}}^{\text{z},\text{p}}\left(1:\text{2,3}\right)\right\}, (\text{z}=\text{1,3},\ldots , N)$$

20 $${\bf {\mathcal B}}_{\text{z}}^{n}=\frac{{\Phi }_{\text{z}}^{n,\text{p}}}{H}\left\{{\bf {\mathcal T}}_{2,n}^{\text{z},\text{p}}\left(1:\text{4,6}\right)-\left[{\bf {\mathcal T}}_{2,n}^{\text{z},\text{p}}\left(\text{6,6}\right)/{\bf {\mathcal T}}_{2,n}^{\text{z},\text{p}}\left(\text{6,5}\right)\right]{\bf {\mathcal T}}_{2,n}^{\text{z},\text{p}}\left(1:\text{4,5}\right)\right\},$$

where the essential input modal piezoelectric electric potentials, ${\Phi }_{\text{z}}^{n,\text{p}} \left(n=\text{0,1},\text{2,3},\ldots \right),$ will be derived in the controller design “Controller design”.

### Fluid/structure interaction (FSI)

The undetermined modal parameters $({p}_{1n}^{\text{scat}}$, ${p}_{2n}^{\text{trans}}{;\overline{\mathbf{V}} }_{\text{2,0}}, {\overline{\mathbf{V}} }_{2,n})$ should be obtained by enforcing the appropriate external/internal multi-physic boundary conditions at the outer/inner interfaces of the hybrid multimorph spherical shell transducer with the neighboring fluids. Therefore, continuousness of structural and normal fluid velocities, fluid pressure and normal stress, alongside nonexistence of tangential stress at $r={R}_{\text{in}},{R}_{\text{ex}}$ require^79^:

$$\left(-\text{i}\omega \right) W{|}_{r={R}_{\text{in}},{R}_{\text{ex}}}={v}_{r}{|}_{r={R}_{\text{in}},{R}_{\text{ex}}}=-{\left[\frac{\partial \left({p}_{1}^{\text{inc}}+{p}_{1}^{\text{scat}}\right)}{\partial r}\right]}_{r={R}_{\text{in}},{R}_{\text{ex}}},$$

21 $$\frac{1}{r}{\Sigma }_{rr}{|}_{r={R}_{\text{in}},{R}_{\text{ex}}}=-p{|}_{r={R}_{\text{in}},{R}_{\text{ex}}}, {\Sigma }_{r\theta }{|}_{r={R}_{\text{in}},{R}_{\text{ex}}}=0,$$

which after direct implementation of the global state space solution (Eq. 17) along with the associated sound field expansions (Eq. 2) result in the following two final decoupled sets of matrix algebraic equations:

$$\left[\begin{array}{cccc}-\text{i}\omega {R}_{\text{in}}{\bf {\mathcal G}}_{0}\left(\text{2,1}\right)& -\text{i}\omega {R}_{\text{in}}{\bf {\mathcal G}}_{0}\left(\text{2,2}\right)& {h}_{0}^{{{\prime}}}\left({k}_{\text{ex}}{R}_{\text{ex}}\right)& 0\\ 0& -\text{i}\omega {R}_{\text{in}}& 0& {j}_{0}^{{{\prime}}}\left({k}_{\text{in}}{R}_{\text{in}}\right)\\ \frac{{R}_{\text{in}}{C}_{44}^{\text{in}}}{{R}_{\text{ex}}}{\bf {\mathcal G}}_{0}\left(\text{1,1}\right)& \frac{{R}_{\text{in}}{C}_{44}^{\text{in}}}{{R}_{\text{ex}}}{\bf {\mathcal G}}_{0}\left(\text{1,2}\right)& -\text{i}\omega {\rho }_{\text{ex}}{h}_{0}\left({k}_{\text{ex}}{R}_{\text{ex}}\right)& 0\\ {C}_{44}^{\text{in}}& 0& 0& -\text{i}\omega {\rho }_{\text{in}}{j}_{0}\left({k}_{\text{in}}{R}_{\text{in}}\right)\end{array}\right]\left[\begin{array}{c}{\Sigma }_{rr}^{0}\\ {W}^{0}\\ {p}_{10}^{\text{scat}}\\ {p}_{20}^{\text{trans}}\end{array}\right]=\left[\begin{array}{c}-{p}_{0}{j}_{0}^{{{\prime}}}\left({k}_{\text{ex}}{R}_{\text{ex}}\right)-{\bf {\mathcal B}}_{0,\text{in}}\left(2\right)-{\bf {\mathcal B}}_{0,\text{ex}}\left(2\right)\\ 0\\ i\omega {\rho }_{\text{ex}}{p}_{0}{j}_{0}\left({k}_{\text{ex}}{R}_{\text{ex}}\right)-{\bf {\mathcal B}}_{0,\text{in}}\left(1\right)-{\bf {\mathcal B}}_{0,\text{ex}}\left(1\right)\\ 0\end{array}\right] , (n=0),$$

$$\left[\begin{array}{cccccc}-\text{i}\omega {R}_{\text{in}}{\bf {\mathcal G}}_{n}\left(\text{4,1}\right)& -\text{i}\omega {R}_{\text{in}}{\bf {\mathcal G}}_{n}\left(\text{4,2}\right)& -\text{i}\omega {R}_{\text{in}}{\bf {\mathcal G}}_{n}\left(\text{4,3}\right)& -\text{i}\omega {R}_{\text{in}}{\bf {\mathcal G}}_{n}\left(\text{4,4}\right)& \left(2n+1\right){\text{i}}^{n}{h}_{n}^{{{\prime}}}\left({k}_{\text{ex}}{R}_{\text{ex}}\right)& 0\\ 0& 0& 0& -\text{i}\omega {R}_{\text{in}}& 0& \left(2n+1\right){\text{i}}^{n}{j}_{n}^{{{\prime}}}\left({k}_{\text{in}}{R}_{\text{in}}\right)\\ \frac{{R}_{\text{in}}{C}_{44}^{\text{in}}}{{R}_{\text{ex}}}{\bf {\mathcal G}}_{n}\left(\text{1,1}\right)& \frac{{R}_{\text{in}}{C}_{44}^{\text{in}}}{{R}_{\text{ex}}}{\bf {\mathcal G}}_{n}\left(\text{1,2}\right)& \frac{{R}_{\text{in}}{C}_{44}^{\text{in}}}{{R}_{\text{ex}}}{\bf {\mathcal G}}_{n}\left(\text{1,3}\right)& \frac{{R}_{\text{in}}{C}_{44}^{\text{in}}}{{R}_{\text{ex}}}{\bf {\mathcal G}}_{n}\left(\text{1,4}\right)& -\omega {\rho }_{\text{ex}}\left(2n+1\right){\text{i}}^{n+1}{h}_{n}\left({k}_{\text{ex}}{R}_{\text{ex}}\right)& 0\\ {C}_{44}^{\text{in}}& 0& 0& 0& 0& -\omega {\rho }_{\text{in}}\left(2n+1\right){\text{i}}^{n+1}{j}_{n}\left({k}_{\text{in}}{R}_{\text{in}}\right)\\ {\bf {\mathcal G}}_{n}\left(\text{2,1}\right)& {\bf {\mathcal G}}_{n}\left(\text{2,2}\right)& {\bf {\mathcal G}}_{n}\left(\text{2,3}\right)& {\bf {\mathcal G}}_{n}\left(\text{2,4}\right)& 0& 0\\ 0& 0& 0& 0& 0& 1\end{array}\right]\left[\begin{array}{c}{\Sigma }_{rr}^{n}\\ {\Sigma }_{2}^{n}\\ {G}^{n}\\ {W}^{n}\\ {p}_{1n}^{\text{scat}}\\ {p}_{2n}^{\text{trans}}\end{array}\right]$$

22 $$=\left[\begin{array}{c}-{p}_{0}\left(2n+1\right){\text{i}}^{n}{j}_{n}^{{{\prime}}}\left({k}_{\text{ex}}{R}_{\text{ex}}\right)-{\bf {\mathcal B}}_{n,\text{in}}\left(4\right)-{\bf {\mathcal B}}_{n,\text{ex}}\left(4\right)\\ 0\\ \omega {\rho }_{\text{ex}}{p}_{0}\left(2n+1\right){\text{i}}^{n+1}{j}_{n}\left({k}_{\text{ex}}{R}_{\text{ex}}\right)-{\bf {\mathcal B}}_{n,\text{in}}\left(1\right)-{\bf {\mathcal B}}_{n,\text{ex}}\left(1\right)\\ 0\\ -{\bf {\mathcal B}}_{n,\text{in}}\left(2\right)-{\bf {\mathcal B}}_{n,\text{ex}}\left(2\right)\\ 0\end{array}\right] . (n\ge 1),$$

### Controller design

Now, a multiple-input multiple-output active damping control (MIMO-ADC) scheme^80,81^ based on the positive/negative position/velocity feedback (PPF/NVF) control strategy can be adopted here for operative mitigation of the acousto-elastic response of the hybrid smart multimorph structure. Accordingly, one should consider the following two sets of second order compensators established on the modal components of input radial velocities and displacements of the viscoelastic and piezoelectric laminae in the forms (see Fig. 1):

$${\ddot{\eta }}_{\text{Pos},\text{z}}^{n,\text{p}}+2{\varsigma }_{0,\text{z}}^{n,\text{p}}{\omega }_{0,\text{z}}^{n,\text{p}}{\dot{\eta }}_{\text{Pos},\text{z}}^{n,\text{p}}+{({\omega }_{0,\text{z}}^{n,\text{p}})}^{2}{\eta }_{\text{Pos},\text{z}}^{n,\text{p}}={\left({\omega }_{0,\text{z}}^{n,\text{p}}\right)}^{2}{W}_{\text{z}}^{n}\left(\xi ={\xi }_{\text{z},\text{p}}^{{q}_{\text{p}}^{\text{z}}},\text{0,0},t\right),$$

$${\ddot{\eta }}_{\text{Vel},\text{z}}^{n,\text{p}}+2{\varsigma }_{0,\text{z}}^{n,\text{p}}{\omega }_{0,\text{z}}^{n,\text{p}}{\dot{\eta }}_{\text{Vel},\text{z}}^{n,\text{p}}+{({\omega }_{0,\text{z}}^{n,\text{p}})}^{2}{\eta }_{\text{Vel},\text{z}}^{n,\text{p}}={\left({\omega }_{0,\text{z}}^{n,\text{p}}\right)}^{2}{\dot{W}}_{\text{z}}^{n}\left(\xi ={\xi }_{\text{z},\text{p}}^{{q}_{\text{p}}^{\text{z}}},\text{0,0},t\right), \left(z=1, 3,\ldots ,N\right),$$

$${\ddot{\eta }}_{\text{Pos},\text{z}}^{n,\text{v}}+2{\varsigma }_{0,\text{z}}^{n,\text{v}}{\omega }_{0,\text{z}}^{n,\text{v}}{\dot{\eta }}_{\text{Pos},\text{z}}^{n,\text{v}}+{({\omega }_{0,\text{z}}^{n,\text{v}})}^{2}{\eta }_{\text{Pos},\text{z}}^{n,\text{v}}={\left({\omega }_{0,\text{z}}^{n,\text{v}}\right)}^{2}{w}_{\text{z}}^{n}\left(\xi ={\xi }_{\text{z},\text{v}}^{{q}_{\text{v}}^{\text{z}}},\text{0,0},t\right),$$

23 $${\ddot{\eta }}_{\text{Vel},\text{z}}^{n,\text{v}}+2{\varsigma }_{0,\text{z}}^{n,\text{v}}{\omega }_{0,\text{z}}^{n,\text{v}}{\dot{\eta }}_{\text{Vel},\text{z}}^{n,\text{v}}+{({\omega }_{0,\text{z}}^{n,\text{v}})}^{2}{\eta }_{\text{Vel},\text{z}}^{n,\text{v}}={\left({\omega }_{0,\text{z}}^{n,\text{v}}\right)}^{2}{\dot{w}}_{\text{z}}^{n}\left(\xi ={\xi }_{\text{z},\text{v}}^{{q}_{\text{v}}^{\text{z}}},\text{0,0},t\right), \left(z=2, 4,\ldots ,N-1\right),$$

Where $({\eta }_{\text{Pos},\text{z}}^{n,\text{p}},{\eta }_{\text{Vel},\text{z}}^{n,\text{p}} ;{W}_{\text{z}}^{n},{\dot{W}}_{\text{z}}^{n})$ refer to the position/velocity modal controller coordinates and displacement/velocity feedback of the piezoelectric layers, while $\left({\eta }_{\text{Pos},\text{z}}^{n,\text{v}},{\eta }_{\text{Vel},\text{z}}^{n,\text{v}}; {w}_{\text{z}}^{n},{\dot{w}}_{\text{z}}^{n}\right)$ signify the position/velocity modal controller coordinates and displacement/velocity feedback of the SVE layers, respectively, and $({\varsigma }_{0,\text{z}}^{n,\text{p}},{\varsigma }_{0,\text{z}}^{n,\text{v}} ;{\omega }_{0,\text{z}}^{n,\text{p}},{\omega }_{0,\text{z}}^{n,\text{v}})$ are the associated modal controller damping ratio and natural frequency. For harmonic oscillations, one can conveniently express the above parameters in the standard forms:

$${W}_{z}^{n}\left(\xi ,\text{0,0},t\right)={\overline{W} }_{z}^{n}\left(\xi ,\text{0,0},\omega \right){e}^{\text{i}\omega t},$$

$${\eta }_{\text{Pos},z}^{n,p}\left(t\right)={\overline{\eta }}_{\text{Pos},z}^{n,p}\left(\omega \right){e}^{\text{i}\omega t}, {\eta }_{\text{Vel},z}^{n,p}\left(t\right)={\overline{\eta }}_{\text{Vel},z}^{n,p}\left(\omega \right){e}^{\text{i}\omega t}, ( z=1, 3,\ldots ,N),$$

$${w}_{z}^{n}\left(\xi ,\text{0,0},t\right)={\overline{w} }_{z}^{n}\left(\xi ,\text{0,0},\omega \right){e}^{\text{i}\omega t},$$

24 $${\eta }_{\text{Pos},z}^{n,v}\left(t\right)={\overline{\eta }}_{\text{Pos},z}^{n,v}{\left(\omega \right)e}^{\text{i}\omega t}, {\eta }_{\text{Vel},z}^{n,v}\left(t\right)={\overline{\eta }}_{\text{Vel},z}^{n,v}{\left(\omega \right)e}^{\text{i}\omega t}, \left(z=2, 4,\ldots ,N-1\right).$$

where by using the above transformations in modal coordinate Eqs. (23), one obtains the frequency-dependent forms of controller coordinates:

$${\overline{\eta }}_{\text{Pos},\text{z}}^{n,\text{p}}\left(\omega \right)=\left[\frac{{\left({\omega }_{0,\text{z}}^{n,\text{p}}\right)}^{2}}{\left({\omega }_{0,\text{z}}^{n,\text{p}}-{\omega }^{2}\right)+2\text{i}\omega {\varsigma }_{0,\text{z}}^{n,\text{p}}{\omega }_{0,\text{z}}^{n,\text{p}}}\right]{\overline{W} }_{\text{z}}^{n}\left({\xi }_{\text{z},\text{p}}^{{q}_{\text{p}}^{\text{z}}},\text{0,0},\omega \right),$$

$${\overline{\eta }}_{\text{Vel},z}^{n,p}\left(\omega \right)=\left[\frac{-\text{i}\omega {\left({\omega }_{0,\text{z}}^{n,\text{p}}\right)}^{2}}{\left({\omega }_{0,\text{z}}^{n,\text{p}}-{\omega }^{2}\right)+2\text{i}\omega {\varsigma }_{0,\text{z}}^{n,\text{p}}{\omega }_{0,\text{z}}^{n,\text{p}}}\right]{\overline{W} }_{\text{z}}^{n}\left({\xi }_{\text{z},\text{p}}^{{q}_{\text{p}}^{\text{z}}},\text{0,0},\omega \right),(z=1, 3,\ldots ,N),$$

$${\overline{\eta }}_{\text{Pos},z}^{n,v}\left(\omega \right)=\left[\frac{{\left({\omega }_{0,\text{z}}^{n,\text{v}}\right)}^{2}}{\left({\omega }_{0,\text{z}}^{n,\text{v}}-{\omega }^{2}\right)+2\text{i}\omega {\varsigma }_{0,\text{z}}^{n,\text{v}}{\omega }_{0,\text{z}}^{n,\text{v}}}\right]{\overline{w} }_{z}^{n}\left({\xi }_{\text{z},\text{v}}^{{q}_{\text{v}}^{\text{z}}},\text{0,0},\omega \right),$$

25 $${\overline{\eta }}_{\text{Vel},z}^{n,v}\left(\omega \right)=\left[\frac{-\text{i}\omega {\left({\omega }_{0,\text{z}}^{n,\text{v}}\right)}^{2}}{\left({\omega }_{0,\text{z}}^{n,\text{v}}-{\omega }^{2}\right)+2\text{i}\omega {\varsigma }_{0,\text{z}}^{n,\text{v}}{\omega }_{0,\text{z}}^{n,\text{v}}}\right]{\overline{w} }_{z}^{n}\left({\xi }_{\text{z},\text{v}}^{{q}_{\text{v}}^{\text{z}}},\text{0,0},\omega \right).( z=2, 4,\ldots ,N-1),$$

Next, by following the standard ADC strategy^82^, the above modal controller coordinates can be amplified through the associated gain parameters $\left({G}_{\text{Pos},\text{z}}^{n,\text{p}},{G}_{Vel,\text{z}}^{n,\text{p}} ; {G}_{\text{Pos},\text{z}}^{n,\text{v}},{G}_{Vel,\text{z}}^{n,\text{v}}\right)$ that are input into the corresponding piezoelectric and SVE actuator layers as:

$${\Phi }_{\text{z}}^{n,\text{p}}\left({\xi }_{\text{z},\text{p}}^{1}\right) =0,$$

$${\Phi }_{\text{z}}^{n,\text{p}}\left({\xi }_{\text{z},\text{p}}^{{q}_{\text{p}}^{\text{z}}}\right)={G}_{\text{Pos},\text{z}}^{n,\text{p}}{\left({\omega }_{0,\text{z}}^{n,\text{p}}\right)}^{2}{\overline{\eta }}_{\text{Pos},\text{z}}^{n,\text{p}}-{G}_{\text{Vel},\text{z}}^{n,\text{p}}{\left({\omega }_{0,\text{z}}^{n,\text{p}}\right)}^{2}{\overline{\eta }}_{\text{Vel},z}^{n,p}, \left(z=1, 3,\ldots ,N\right),$$

$${\Phi }_{\text{z}}^{n,\text{v}}\left({\xi }_{\text{z},\text{v}}^{1}\right) =0,$$

26 $${\Phi }_{\text{z}}^{n,\text{v}}\left({\xi }_{\text{z},\text{v}}^{{q}_{\text{v}}^{\text{z}}}\right)={G}_{\text{Pos},\text{z}}^{n,\text{v}}{\left({\omega }_{0,\text{z}}^{n,\text{v}}\right)}^{2}{\overline{\eta } }_{\text{Pos},\text{z}}^{\text{n},\text{v}}-{G}_{\text{Vel},\text{z}}^{n,\text{v}}{\left({\omega }_{0,\text{z}}^{n,\text{v}}\right)}^{2}{\overline{\eta }}_{\text{Vel},z}^{n,v},(z=2, 4,\ldots ,N-1),$$

which after application of modal coordinate transformation Eqs. (25), reduces to

$${\Phi }_{\text{z}}^{n,\text{p}}\left({\xi }_{\text{z},\text{p}}^{1}\right) =0,$$

$${\Phi }_{\text{z}}^{n,\text{p}}\left({\xi }_{\text{z},\text{p}}^{{q}_{\text{p}}^{\text{z}}}\right)=\left({k}_{\text{Pos},\text{z}}^{n,\text{p}}+{k}_{\text{Vel},\text{z}}^{n,\text{p}}\right){\overline{W} }_{\text{z}}^{n}\left({\xi }_{\text{z},\text{p}}^{{q}_{\text{p}}^{\text{z}}},\text{0,0}\right),(z=1, 3,\ldots ,N),$$

$${\Phi }_{\text{z}}^{n,\text{v}}\left({\xi }_{\text{z},\text{v}}^{1}\right) =0,$$

27 $${\Phi }_{\text{z}}^{n,\text{v}}\left({\xi }_{\text{z},\text{v}}^{{q}_{\text{v}}^{\text{z}}}\right)=\left({k}_{\text{Pos},\text{z}}^{n,\text{v}}+{k}_{\text{Vel},\text{z}}^{n,\text{v}}\right){\overline{w} }_{z}^{n}\left({\xi }_{\text{z},\text{v}}^{{q}_{\text{v}}^{\text{z}}},\text{0,0}\right), \left(z=2, 4,\ldots ,N-1\right),$$

where

$${k}_{\text{Pos},\text{z}}^{n,\text{p}}=\left[\frac{{G}_{\text{Pos},\text{z}}^{n,\text{p}}{\left({\omega }_{0,\text{z}}^{n,\text{p}}\right)}^{4}}{\left({\omega }_{0,\text{z}}^{n,\text{p}}-{\omega }^{2}\right)+2\text{i}{\omega \varsigma }_{0,\text{z}}^{n,\text{p}}{\omega }_{0,\text{z}}^{n,\text{p}}}\right],$$

$${k}_{\text{Pos},\text{z}}^{n,\text{v}}=\left[\frac{{G}_{\text{Pos},\text{z}}^{n,\text{v}}{\left({\omega }_{0,\text{z}}^{n,\text{v}}\right)}^{4}}{\left({\omega }_{0,\text{z}}^{n,\text{v}}-{\omega }^{2}\right)+2\text{i}{\omega \varsigma }_{0,\text{z}}^{n,\text{v}}{\omega }_{0,\text{z}}^{n,\text{v}}}\right], {k}_{\text{Vel},\text{z}}^{n,\text{p}}=-\left[\frac{-\text{i}\omega {G}_{\text{Vel},\text{z}}^{n,\text{p}}{\left({\omega }_{0,\text{z}}^{n,\text{p}}\right)}^{4}}{\left({\omega }_{0,\text{z}}^{n,\text{p}}-{\omega }^{2}\right)+2\text{i}\omega {\varsigma }_{0,\text{z}}^{n,\text{p}}{\omega }_{0,\text{z}}^{n,\text{p}}}\right],$$

$${k}_{\text{Vel},\text{z}}^{n,\text{v}}=-\left[\frac{-\text{i}\omega {G}_{\text{Vel},\text{z}}^{n,\text{v}}{\left({\omega }_{0,\text{z}}^{n,\text{v}}\right)}^{4}}{\left({\omega }_{0,\text{z}}^{n,\text{v}}-{\omega }^{2}\right)+2\text{i}\omega {\varsigma }_{0,\text{z}}^{n,\text{v}}{\omega }_{0,\text{z}}^{n,\text{v}}}\right],$$

## Results and discussion

**Figure 2:**
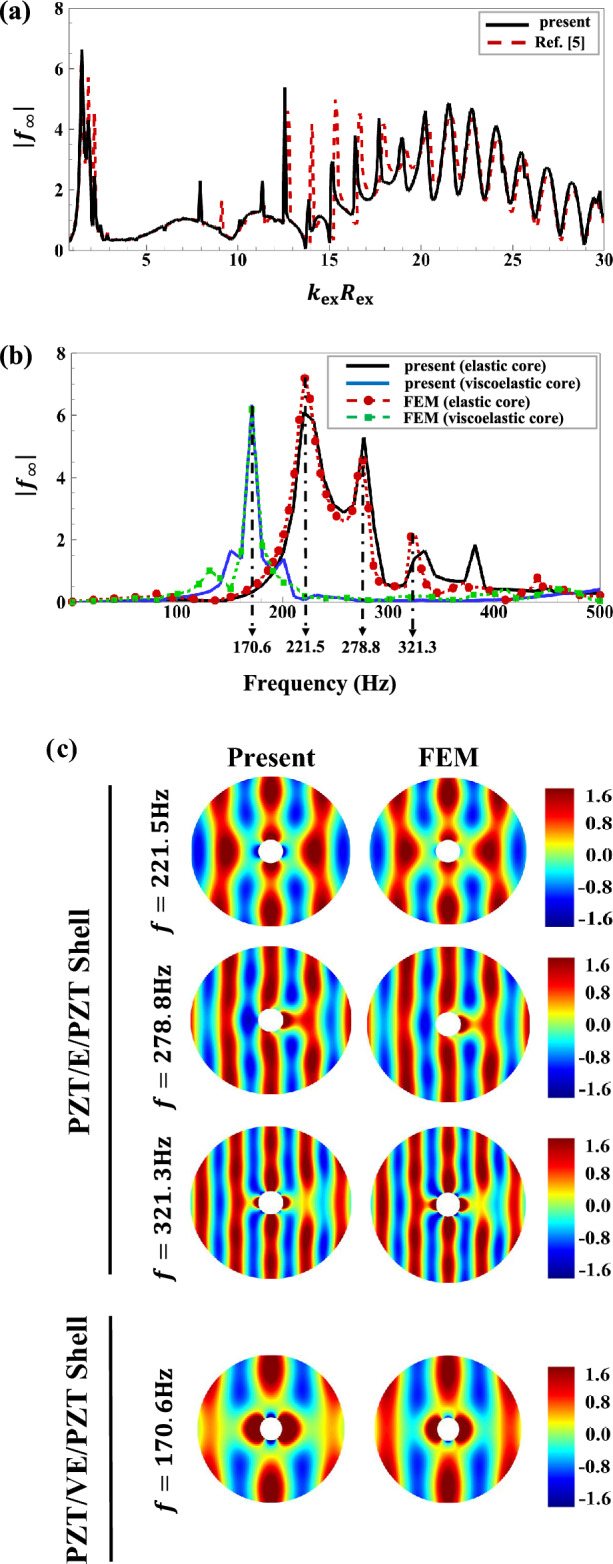
**(a)** Far-field backscattering form function amplitude spectrum for incident plane wave scattering from a water-submerged and air-filled thick-walled aluminum spherical shell. **(b)** Far-field backscattering form function amplitude spectrums for water-submerged and air-filled three layered PZT/E/PZT and PZT/VE/PZT shells. **(c)** Near-field acoustic pressure directivity patterns for the piezo-sandwich spherical shells at the major resonance peaks detected in the $\left|{f}_{\infty }\left(\theta =\pi ,{k}_{\text{ex}}{R}_{\text{ex}}\right)\right|$ spectrum of Fig. 2b.

Noting the relatively large number of input parameters present in the above FSI analysis, while keeping in mind our computing limitations, a number of particular simulations will be discussed in this section. However, before offering the principal simulation results, we should shortly confirm the general validity of the proposed formulation. In particular, the main developed code will be first employed to compute the back-scattered form function spectrum in the far-field based on the standard formula^83^:

28 $$\left|{f}_{\infty }\left(\theta =\pi ,{k}_{\text{ex}}{R}_{\text{ex }}\right)\right|=\left|\frac{2}{\text{i}{k}_{\text{ex}}{R}_{\text{ex }}}\sum_{n=0}^{\infty }(2n+1){p}_{1n}^{\text{scat}}{P}_{n}(\text{cos}\theta )\right|,$$

for incident plane wave scattering from a water-submerged and air-filled thick-walled aluminum spherical shell transducer with the physical and geometrical properties as given in the first part of Table 1. The analytical results, as shown in Fig. 2a, exhibit relatively good accordance with the benchmark (experimental) results in Ref.^5^ for a wide range of non-dimensional incident wave frequencies $({0<k}_{\text{ex}}{R}_{\text{ex }}\le 30)$. Convergence of analytical results was secured through a general trial/error manner that includes gradual addition of the truncation constants in the infinite series solutions while looking for consistency of the calculated numerical results. It was found that using up to two hundred modes $\left({n}_{\text{max}}=200\right)$ can yield adequate numerical outputs in the considered frequency range. As a further verification, we calculated $\left|{f}_{\infty }\left(\theta =\pi ,{k}_{\text{ex}}{R}_{\text{ex}}\right)\right|$ for water-submerged and air-filled three layered piezo-sandwich PZT/E/PZT and PZT/VE/PZT shells as a function of frequency $(0<f\le 500\text{Hz})$ with the associated material/geometrical parameters as provided in Table 1. The outcome, as illustrated in Fig. 2b, reveal good agreements with those simulated by coupling the “Solid Mechanics,” “Electrostatics,” and “Pressure Acoustics” modules of COMSOL package (v5.4). The growing discrepancies observed beyond $f=300\text{Hz}$ are evidently explained by the intrinsic limitations of the conventional FEM approach, keeping in mind the fairly good broadband accordance already obtained between the experimental and analytical results in Fig. 2a. Further improvements in the FEM results at higher frequencies could certainly be achieved by further elaborations on the adopted the FEM meshing strategy (e.g., grid spacing, number/type of elements, etc.) which is accompanied by increased computational costs. Also, in the latter simulations, the linear viscoelastic behavior of core material is based on the standard Zener model^84^ with the associated complex (frequency-dependent) Lame parameters described as:${\lambda }_{\text{v}}^{*}=2{\mu }_{\text{v}}^{*}{\upsilon }_{\text{v}}/\left(1-2{\upsilon }_{\text{v}}\right), {\mu }_{\text{v}}^{*}={\mu }_{\text{v}}{\prime}+\text{i}{\mu }_{\text{v}}^{{\prime}{\prime}},$ in which^85^:

**Table 1:** Input material and geometrical parameters.

Elastic spherical shell	
Elastic material $\lambda$ = 54.4 $\text{GPa}$, $\mu$= 26.7 $\text{GPa}$, $\rho$= 2790 $\text{kg}/{\text{m}}^{3}$ Geometric parameters ${R}_{\text{in}}=0.95 \text{m}$, ${R}_{\text{ex}}=1 \text{m}$, ${H}_{\text{v}}\approx 0.05 \text{m},$ ${H}_{\text{p}}\approx 0 \text{m},$ *N* = 3	Ambient acoustic fluids ${\rho }_{\text{in}}=1.2\text{kg}/{\text{m}}^{3},$ ${\rho }_{\text{ex}}=1000\text{kg}/{\text{m}}^{3},$ ${c}_{\text{in}}=343\text{m}/\text{s} ,$ ${c}_{\text{ex}}=1410\text{m}/\text{s},$ ${p}_{0}=1 \text{Pa}$
PZT/E/PZT and PZT/VE/PZT spherical shells	
Piezoelectric (PZT) skin layers ${C}_{11}=138.9\text{GPa}$, ${C}_{12}=77.84\text{GPa}$, ${C}_{13}=74.28\text{GPa}$, ${C}_{33}=115.41\text{GPa}$, ${C}_{44}=25.64\text{GPa}$, ${e}_{15}=12.72\text{C}/{\text{m}}^{2}$, ${e}_{31}=-5.2\text{C}/{\text{m}}^{2}$, ${e}_{33}=15.1\text{C}/{\text{m}}^{2},$ ${\varepsilon }_{11}=762.5\times {10}^{-11} \text{F}/\text{m}$, ${\varepsilon }_{33}=663.2\times {10}^{-11} \text{F}/\text{m}$, ${\rho }_{\text{p}}=7500\text{kg}/{\text{m}}^{3}$	Geometric parameters ${R}_{\text{in}}=1.4 \text{m},$ ${R}_{\text{ex}}=1.5 \text{m},$ ${H}_{\text{p}}=0.02 \text{m},$ ${H}_{\text{v}}=0.06 \text{m}$
Viscoelastic (VE) core layer	Ambient acoustic fluids
${G}_{\text{v}}=10 \text{KPa}$,${G}_{\text{c}}=50 \text{KPa},$ ${\tau }_{\text{v}}={10}^{-4} \text{s},$ ${\rho }_{\text{v}}=1300 \text{kg}/{\text{m}}^{3},$ ${\upsilon }_{\text{v}}=0.49$	${\rho }_{\text{in}}=1.2\text{kg}/{\text{m}}^{3},$ ${\rho }_{\text{ex}}=1000\text{kg}/{\text{m}}^{3},$ ${c}_{\text{in}}=343\text{m}/\text{s} ,$ ${c}_{\text{ex}}=1410\text{m}/\text{s},$ ${p}_{0}=1\text{Pa}$
Elastic (E) core layer	
${\lambda }_{\text{v}}$=40.38 $\text{GPa}$, ${\mu }_{\text{v}}$=26.92 $\text{GPa}$, ${\rho }_{\text{v}}$=2700 $\text{kg}/{\text{m}}^{3}$	
FEM mesh Piezoelectric skin layers: 42,000 free tetrahedral elements Viscoelastic core layer: 18,000 free tetrahedral elements Elastic core layer: 18,000 free tetrahedral elements Internal/external acoustic mediums: 140,000 free tetrahedral elements Perfectly Matched Layer (PML): 35,000 free tetrahedral elements Maximum element size $\le \left({\lambda }_{\text{max}}^{\text{in},\text{ex}}/6={c}_{\text{in},\text{ex}}/6f\right)$

$${\mu }_{\text{v}}{\prime}={G}_{\text{c}}+{G}_{\text{v}}\left\{{\left(\upomega {\tau }_{\text{v}}\right)}^{2}/[1+{\left(\upomega {\tau }_{\text{v}}\right)}^{2}\right]\},$$

29 $${\mu }_{\text{v}}^{{\prime}{\prime}}={G}_{\text{v}}\left\{\left(\upomega {\tau }_{\text{v}}\right)/\left[1+{\left(\upomega {\tau }_{\text{v}}\right)}^{2}\right]\right\},$$

where the numerical values of viscoelastic parameters $({G}_{\text{c}}$,${G}_{\text{v}}, {\tau }_{\text{v}} )$ as well as the FEM meshing information are provided at the end of Table 1. Lastly, Fig. 2c compares the total resultant 2D acoustic pressure directivity contours (i.e., ${p}_{1}^{\text{tot}}={p}_{1}^{\text{inc}}+{p}_{1}^{\text{scat}}$ with ${p}_{0}=1\text{Pa})$ for the piezo-sandwich spherical shell configurations of Fig. 2b (i.e., PZT/E/PZT and PZT/VE/PZT shells) based on the present exact 3D elasticity theory and the FEM approach at selected incident wave frequencies associated with the major resonance peaks detected in the $\left|{f}_{\infty }\left(\theta =\pi ,{k}_{\text{ex}}{R}_{\text{ex}}\right)\right|$ spectrum (i.e., at $f\approx 170.6\text{Hz}$ for the piezo-sandwich shell with a viscoelastic core, and at $f\approx 221.5, 278.8, 321.3\text{Hz}$ for the piezo-sandwich shell with an elastic core, as marked in Fig. 2b). Good agreements are obtained as illustrated in the figure. Here, it should be noted that for a better comparison, the maximum calculated total resultant acoustic pressure magnitude $(\text{i}.\text{e}., \left|{p}_{1}^{\text{tot}}\right|=1.6\text{Pa})$ in all circular computational domains $(\left|r/{R}_{\text{ex}}\right|\le 10/1.5=6.67)$ has been adopted for setting the single scale limit of the associated color bars.

**Figure 3:**
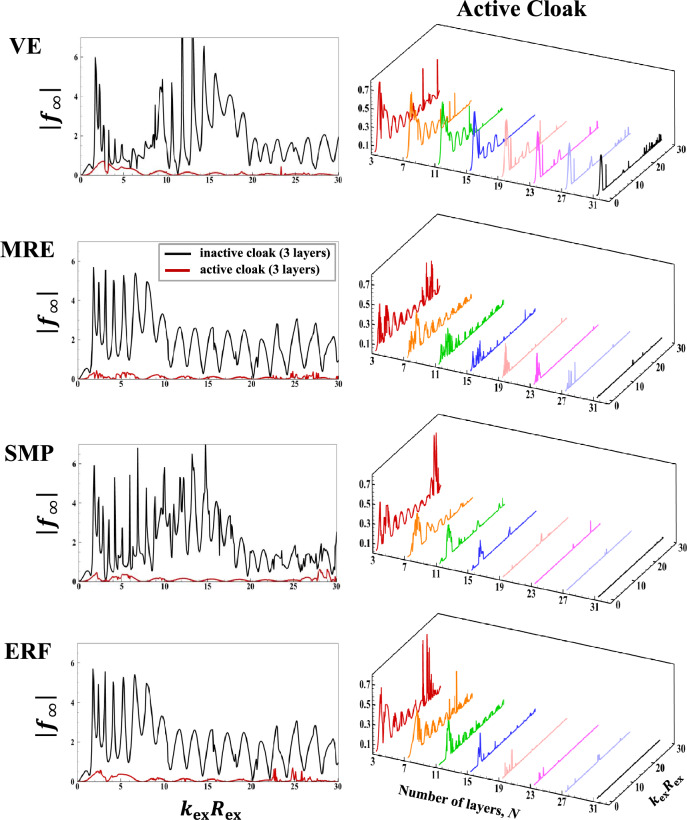
Inactive and active far-field backscattering form function spectrums for the smart hybrid bimorph and multimorph piezoelectric spherical shell transducers for four distinct functional viscoelastic core materials.

The subplots in the first column of Fig. 3 present the inactive and active far-field backscattering form function spectrum subplots, $\left|{f}_{\infty }\left(\theta =\pi ,{k}_{\text{ex}}{R}_{\text{ex }}\right)\right|,$ for the smart hybrid water-submerged and air-filled thick-walled $\left({\frac{{R}_{\text{in }}}{{R}_{\text{ex }}}}=\frac{0.825\text{m}}{1.1\text{m}}=0.75;25\% \text{shell}\right)$ bimorph (parallel-connected) sandwich piezoelectric spherical shells (*N* = 3) with four selected functional viscoelastic core materials, namely, conventional (inactive) viscoelastic material (VE), smart magnetorheological elastomer (MRE), smart shape memory polymer (SMP), and smart electrorheological fluid (ERF), as respectively described in Supplementary Material B (see Eqs. B-1 to B-4). Similarly, the subplots in the second column of Fig. 3 display the active far-field backscattering form function spectrum subplots for the thick-walled $\left({\frac{{R}_{\text{in }}}{{R}_{\text{ex }}}}=\frac{0.825\text{m}}{1.1\text{m}}=0.75\right)$ smart hybrid multimorph piezoelectric spherical shell cloaks (${q}_{\text{p}}^{\text{z}}=10,z=\text{1,3},\ldots ,N;{q}_{\text{v}}^{\text{z}}=30,z=\text{2,4},\ldots ,N-1 ;N=\text{3,7},\text{11,15,19,23,27,31} ;\text{see Fig}. 1$) with the following core layer materials: conventional (inactive) viscoelastic (VE), semi-active magnetorheological elastomeric (MRE), semi-active shape memory polymer (SMP), and semi-active electrorheological fluid (ERF), as briefly described in Supplementary Material B (and the remaining input parameters as provided in Table 1). Here, it should be noted that the Particle Swarm Optimization (PSO) algorithm^86^ was utilized in all simulations for tuning various modal parameters of the adopted ADC controller based on locations and velocities of $\overline{k }-\text{th}$ particle at $\overline{m }-\text{th}$ generation^87^ with $\left|{f}_{\infty }\left(\theta =\pi ,{k}_{\text{ex}}{R}_{\text{ex}}\right)\right|$ serving as the key optimization cost function and with the following lower and upper bounds of the optimization parameters: ${L}_{\text{b}}=\left[{10}^{-14}, {10}^{-17}, 0.5\omega , 0.1\right]$, ${U}_{\text{b}}=\left[{10}^{-9}, {10}^{-11}, \omega , 0.45\right].$ The key observations are as follows. First, in case of the bimorph sandwich cloak (*N* = 3), the substantial suppression effects of the hybrid PZT/VE and PZT/SVE configurations on the far-field backscattering form functions nearly in the entire range of frequency is clear in the left-side subfigures of Fig. 3 (i.e., note the substantial difference/drop between the black and red colored curves, particularly for the SVE core layers). In particular, one can see a number of relatively wide-band effective cloaking regions (or attenuation bands) predominantly at the high and intermediate frequencies, where the active cloaks yield near-zero backscattering amplitudes, virtually regardless of core layer type. In this regard, the far-field backscattering form functions subplots in the second column subplots of Fig. 3 clearly indicate that by increasing the number of active/semi-active layers, one can effectively increase the width of the these attenuation bands. In particular, one can see wide-band reductions in the backscattering amplitudes for the *N* = 31 for all three multimorph/SVE configurations in the entire frequency spectrum, with the ERF and SMP displaying a slightly better performance compared to MRE.

**Figure 4:**
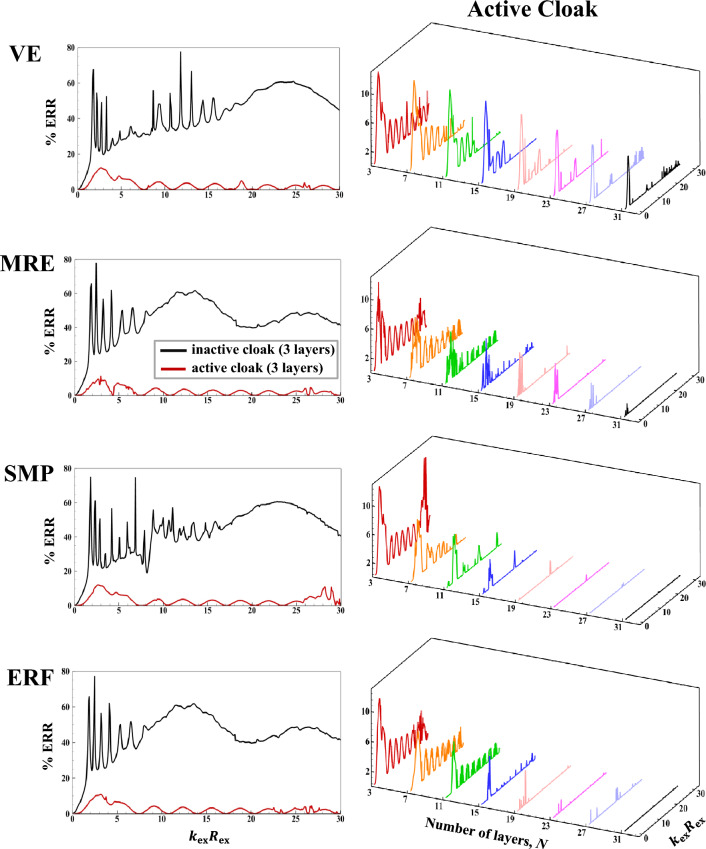
Inactive and active percentage error spectrums for the smart hybrid bimorph and multimorph piezoelectric spherical shell transducers for four distinct functional viscoelastic core materials.

**Figure 5:**
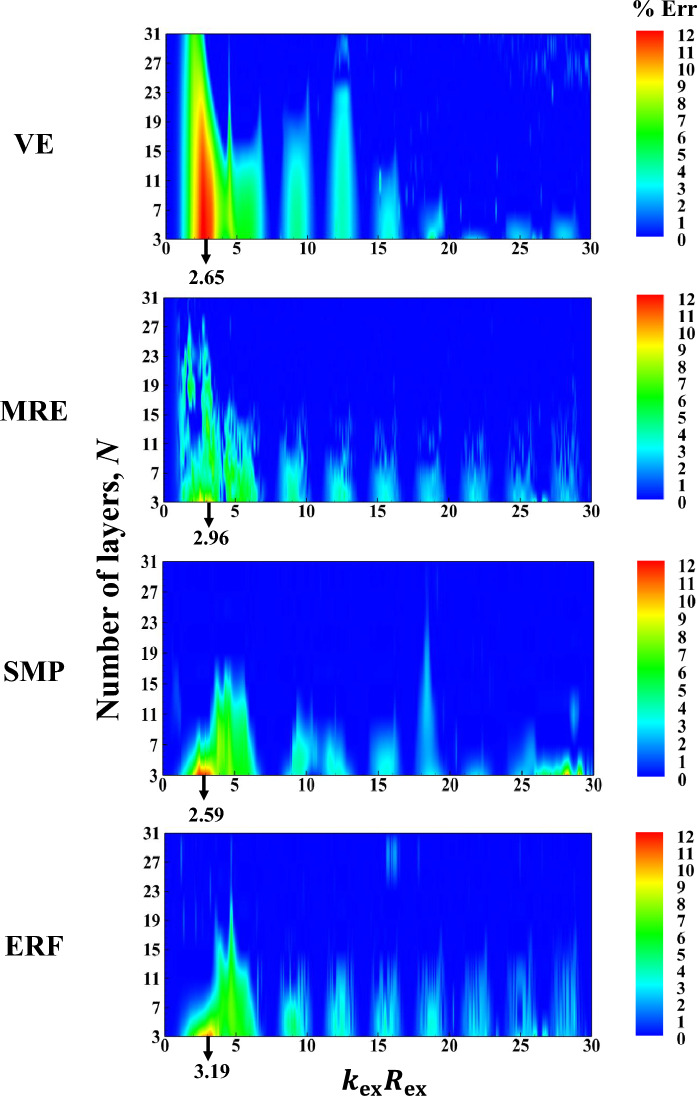
Interpolated active percentage error spectrums for the smart hybrid multimorph piezoelectric spherical shell transducers for four distinct functional viscoelastic core materials.

**Figure 6:**
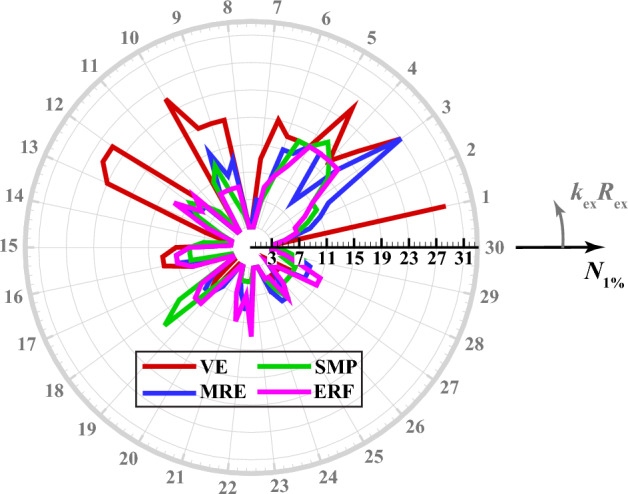
Polar display of data in presented in Table 2 (radial coordinate $r: {N}_{1\text{\%}};$ angular coordinate $\theta : {k}_{\text{ex}}{R}_{\text{ex }}).$

Next, in order to measure the effectiveness of proposed cloaking device, the active cloaked field can be assessed against the background incident pressure field (or the acoustic field without the sphere). In view of that, the error percentage of external pressure field cloaking may advantageously be computed using the following performance index based on $\widehat{N}$*-*measurement locations in the square domain $\left[-5:0.07:5\right] \times \left[-5:0.07:5 \right]{\text{m}}^{2}$ around spherical cloak^88^:

30 $$\%\text{Err}=\frac{\Vert {p}_{1}^{\text{tot}}-{p}_{1}^{\text{inc}}\Vert }{\Vert {p}_{1}^{\text{inc}}\Vert }=\frac{\sqrt{\frac{\sum_{i=1}^{\widehat{N}}{({p}_{1,i}^{\text{tot}}-{p}_{1,i}^{\text{inc}})}^{2}}{\widehat{N}}}}{\sqrt{\frac{\sum_{i=1}^{\widehat{N}}{\left({p}_{1,i}^{\text{inc}}\right)}^{2}}{\widehat{N}}}}\times 100\%$$

where $${p}_{1}^{\text{tot}}={p}_{1}^{\text{inc}}+{p}_{1}^{\text{scat}}.$$ Therefore, in view of above brief description, the subplots in the left half of Fig. 4 present the calculated $$\%\text{Err}$$ spectrums for the smart hybrid sandwich $$(N=3;{q}_{\text{p}}^{\text{z}=1}=10, {q}_{\text{v}}^{\text{z}=2}=30, {q}_{\text{p}}^{\text{z}=3}=10)$$ piezoelectric spherical shells for the selected viscoelastic core materials (see Supplementary Material B). Similarly, the subplots in the right half of Fig. 4 display the calculated $$\%\text{Err}$$ spectrums for the smart hybrid multimorph piezoelectric spherical shell cloaks $$({q}_{\text{p}}^{\text{z}}=10,z=\text{1,3},\ldots ,N;{q}_{\text{v}}^{\text{z}}=30,z=\text{2,4},\ldots ,N-1 ;N=\text{3,7},\text{11,15,19,23,27,31})$$ for the selected viscoelastic core materials. Here, one should note that the $$\%\text{Err}$$ curves associated with $$N=\text{5,9},\text{13,17,21,25,29}$$ are not displayed in the second column subplots of the Fig. 4 in order to increase the clarity. Comments very similar to those made in Fig. 3 can readily be made. The most important distinctions are as follows. Here, one should first note that, in contrast with the inactive backscattering form function subplots in the first column of Fig. 3 (black curves), the $$\%\text{Err}$$ spectrums for the inactive sandwich cloak (*N* = 3) display a clear non-oscillatory amplification effect at the high to intermediate frequencies. The last observation can directly be associated to the increased forward scattering effects in the shadow region of the inactive cloaks. Next, it is clear from the second column subplots of Fig. 4 that the calculated $$\%\text{Err}$$ for the smart hybrid PZT/VE and PZT/SVE multimorph piezoelectric spherical shell cloaks considerably decrease with increasing the number of layers, especially at high incident wave frequencies. In particular, the effect of increasing the number of layers on decreasing the $$\%\text{Err}$$ for the hybrid multimorph cloaks with SVE core layers appear to be much higher than that of the hybrid multimorph cloak with VE core layers, especially in the low to intermediate frequency range. Furthermore, as previously noted in Fig. 3, the hybrid multimorph configurations with ERF and SMP core layers display a slightly better performance compared to that with MRE core layers, particularly in the low to intermediate frequency range. The above observations can better be understood if one makes a cubic spline interpolation^89^ between the above calculated $$\%\text{Err}$$ spectrums for the smart hybrid multimorph piezoelectric spherical shell cloaks $$(N=\text{3,5},\ldots ,\text{29,31})$$ with selected viscoelastic core materials, as presented in the subplots of Fig. 5. Here, a distinct critical cloaking region $$(\%\text{Err}\le 12)$$ is clearly observed in the intermediate frequency range $$({0<k}_{\text{ex}}{R}_{\text{ex }}\le 5)$$ of each bimorph cloaking (*N* = 3) configuration, with its center frequency marked in the associated subplot. Naturally, the cloaking performance of each configuration in this region improves as the number of layers increases roughly to $$\%\text{Err}<4.5$$ for PZT/VE and to $$\%\text{Err}<1$$ for PZT/SVE. Also, one can qualitatively rank the overall cloaking performance of the proposed smart hybrid multimorph configurations in terms of the smallest calculated $$\%\text{Err}$$ in the associated critical regions as follows PZT/SMP, PZT/ERF, PZT/MRE, and PZT/VE. This fact can be more quantitatively observed in Table 2 which tabulates the minimum number of layers in the smart hybrid multimorph cloak $$\left({N}_{1\%}\right)$$ that is required for achieving $$\%\text{Err}<1$$ in a wide range of dimensionless frequencies $$({k}_{\text{ex}}{R}_{\text{ex }}=0.5:0.5:30)$$. It should be noted here that this selected $$\%\text{Err}<1$$ limit is well below the final 2.5% error obtained in a recent study^88^ for a comparable acoustic cloak at its design center frequency. Also, the smallest value of $${{N}_{1\%}}$$ in each tabulated frequency is marked in bold in the associated row of Table 2. Furthermore, the average (rounded) value of $${N}_{1\text{\%}}$$ for each viscoelastic core interlayer in the designated frequency range are listed in the last row of the table. Here, the information given in Table 2 can be better (visually) analyzed when presented in the polar form of Fig. 6. Here, the radial coordinate $$(r)$$ of the plot signifies the minimum number of layers $$({N}_{1\text{\%}})$$ required for achieving $$\%\text{Err}<1,$$ while the angular coordinate $$(\theta )$$ of the plot is set as the dimensionless frequency $$({k}_{\text{ex}}{R}_{\text{ex }}).$$ Comments similar to above remarks can readily be made. The most interesting distinction here is the fact that the overall cloaking performance of the proposed smart hybrid multimorph PZT/VE, PZT/MRE, PZT/ERF, and PZT/SMP configurations in terms of the calculated $${N}_{1\text{\%}}$$ are roughly the same in the lower half (high frequency) region of the polar plot $$\left({15<k}_{\text{ex}}{R}_{\text{ex }}\le 
30\right),$$ while once again one can rank the general cloaking performance in the upper half (low to intermediate frequency) region of the polar plot $$\left({0<k}_{\text{ex}}{R}_{\text{ex }}\le 15\right)$$ as PZT/SVE followed by PZT/VE. The more detailed quantitative comparisons between the various PZT/SVE configurations in each frequency range can be readily made based on the data presented in Table 2 depending on the particular cloaking application where the smallest value of $${N}_{1\text{\%}}$$ has been marked in bold.

**Table 2:** Minimum number of layers in the smart hybrid multimorph cloak $\left({N}_{1\%}\right)$ that is required for achieving $\%\text{Err}<1$ in a wide range of dimensionless frequencies (note that the smallest value of ${N}_{1\text{\%}}$ in each tabulated frequency is marked in boldface).

*K*_ex_*R*_ex_	VE	MRE	SMP	ERF	*K*_ex_*R*_ex_	VE	MRE	SMP	ERF
0.5	**3**	**3**	**3**	**3**	15.5	13	11	**9**	11
1	29	**5**	**5**	**5**	16	13	11	**9**	11
1.5	–	9	**7**	**7**	16.5	9	5	**3**	9
2	–	11	9	**7**	17	**3**	**3**	**3**	**3**
2.5	–	13	11	**9**	17.5	**3**	**3**	**3**	**3**
3	27	27	**11**	**11**	18	**3**	7	13	9
3.5	21	17	**15**	17	18.5	**7**	9	17	11
4	17	**9**	17	17	19	9	9	**7**	11
4.5	25	**17**	19	**17**	19.5	**3**	7	5	5
5	**17**	**17**	**17**	**17**	20	**3**	**3**	**3**	**3**
5.5	17	15	17	**13**	20.5	**3**	**3**	**3**	**3**
6	17	15	**11**	**11**	21	**5**	**5**	**5**	7
6.5	19	7	7	**9**	21.5	**5**	7	**5**	11
7	13	7	**3**	**3**	22	**5**	9	**5**	7
7.5	**3**	**3**	**3**	**3**	22.5	**5**	9	**5**	13
8	**3**	**3**	**3**	**3**	23	**3**	**3**	**3**	**3**
8.5	19	13	**5**	9	23.5	**3**	**3**	**3**	**3**
9	19	11	**7**	9	24	**3**	**3**	**3**	**3**
9.5	19	15	13	9	24.5	7	7	**5**	**5**
10	25	**9**	11	**9**	25	7	9	7	**5**
10.5	**3**	**3**	**3**	**3**	25.5	**5**	9	9	9
11	**3**	**3**	7	**3**	26	**5**	**5**	7	7
11.5	11	**9**	**9**	11	26.5	5	**3**	7	**3**
12	25	**9**	11	**9**	27	**3**	5	7	5
12.5	25	11	**9**	13	27.5	**7**	9	**7**	11
13	23	7	**5**	7	28	**7**	9	**7**	11
13.5	7	**3**	**3**	**3**	28.5	7	9	**5**	7
14	**3**	**3**	**3**	**3**	29	**5**	7	7	**5**
14.5	**3**	**3**	7	**3**	29.5	**3**	**3**	5	**3**
15	11	**9**	**9**	**9**	30	**3**	**3**	**3**	**3**
Average: $(0.5\le {K}_{\text{ex}}{R}_{\text{ex}}\le 15)$	17	11	**9**	**9**	Average: $(15.5\le {K}_{\text{ex}}{R}_{\text{ex}}\le 30)$	**7**	**7**	**7**	**7**

${K}_{\text{ex}}{R}_{\text{ex}}$	VE	MRE	SMP	ERF					
Total average: $(0.5\le {K}_{\text{ex}}{R}_{\text{ex}}\le 30)$	13	**9**	**9**	**9**

**Figure 7:**
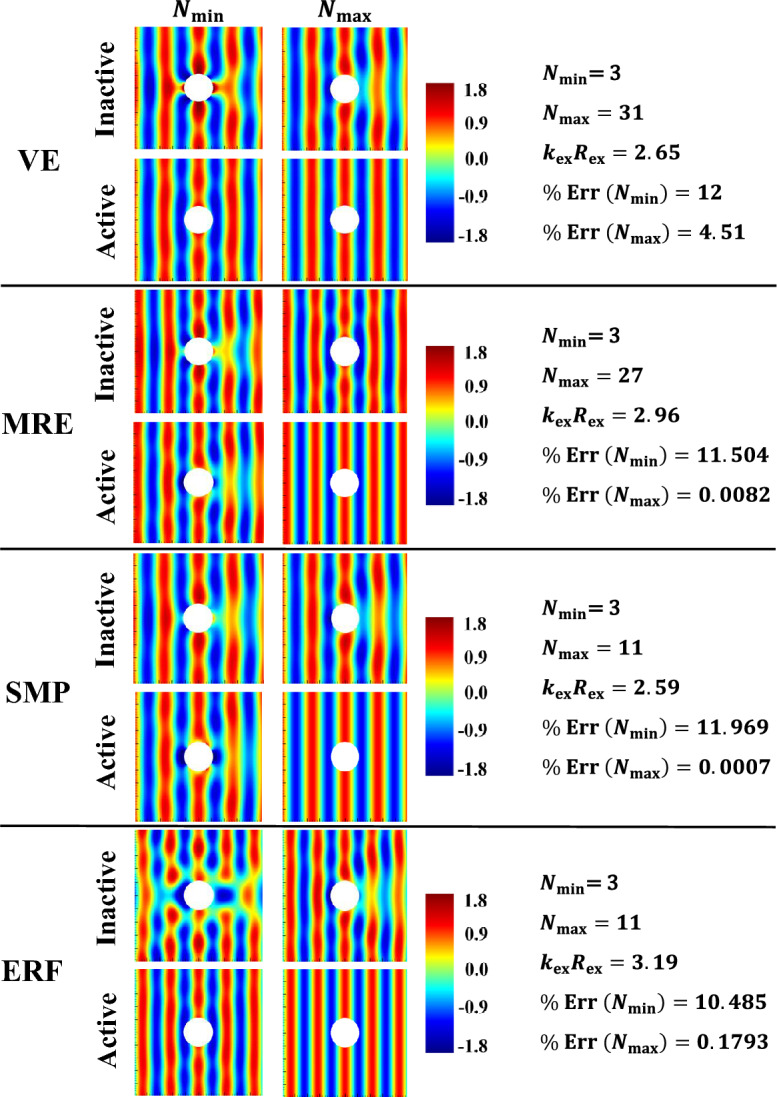
Near-field acoustic pressure directivity patterns for the smart hybrid bimorph and multimorph piezoelectric spherical shell cloaks at the associated critical dimensionless central frequencies.

Now, the total near-field acoustic pressure (i.e., ${p}_{1}^{\text{tot}}={p}_{1}^{\text{inc}}+{p}_{1}^{\text{scat}}$ with ${p}_{0}=1\text{Pa})$ 2D directivity contours presented in the first column subplots of Fig. 7 compare the cloaking performances of the inactive and active hybrid piezo-sandwich cloaks (${N}_{\text{min}}=3$) at the dimensionless central frequencies associated with the critical regions marked in Fig. 5 (i.e., VE: ${k}_{\text{ex}}{R}_{\text{ex}}=2.65,$ MRE: ${k}_{\text{ex}}{R}_{\text{ex}}=2.96,$ SMP: ${k}_{\text{ex}}{R}_{\text{ex}}=2.59,$ ERF: ${k}_{\text{ex}}{R}_{\text{ex}}=3.19)$. Similarly, the total 2D pressure directivity patterns presented in the second column subplots of Fig. 7 are calculated for the active and inactive hybrid multimorph piezoelectric cloaks designed with minimum number of layers required for achieving $\%\text{Err}<1\%$ at the above mentioned dimensionless central frequencies. Here again, the calculated error percentage of external pressure field cloaking $(\%\text{Err})$ are obtained based on $\widehat{N}$*-*measurement locations in the square domain $\left[-5:0.07:5\right] \times \left[-5:0.07:5 \right]{\text{m}}^{2}$ around the spherical cloak. Also, for a better comparison, the maximum total near-field acoustic pressure magnitude $(\text{i}.\text{e}., \left|{p}_{1}^{\text{tot}}\right|=1.8\text{Pa})$ in all calculation domains $(\text{i}.\text{e}., \left|x/{R}_{\text{ex}}\right|,\left|y/{R}_{\text{ex}}\right|\le 5/1.1=5.45)$ has been used for setting the single scale limit of the right hand side color bars. This way, one can better observe the active sound field attenuation effects on the total pressure directivities and magnitudes. Furthermore, one can generally rank the clocking performance of smart hybrid shells (i.e., in terms of both minimum number of cloaking layers and minimum $\%\text{Err}$) at their representative frequency values as follows:

$$\text{PZT}/\text{SMP}: \%\text{Err}\left({N}_{\text{min}}=3\right)=11.969 \text{and} \%\text{Err}\left({N}_{\text{max}}=11\right)=0.0007,$$

$$\text{PZT}/\text{ERF}: \%\text{Err}\left({N}_{\text{min}}=3\right)=10.485 \text{and} \%\text{Err}\left({N}_{\text{max}}=11\right)=0.1793,$$

$$\text{PZT}/\text{MRE}:\%\text{Err}\left({N}_{\text{min}}=3\right)=11.504 \text{and} \%\text{Err}\left({N}_{\text{max}}=27\right)=0.0082,$$

$$\text{PZT}/\text{VE}: \%\text{Err}\left({N}_{\text{min}}=3\right)=12 \text{and} \%\text{Err}\left({N}_{\text{max}}=31\right)=4.51.$$

Moreover, a more detailed examination of the figure reveals that for a low number of smart hybrid cloaking layers $\left({N}_{\text{min}}=3\right)$, a better scattering control is achieved in the backward direction (in comparison with the forward direction), which can be directly linked to adoption of $\left|{f}_{\infty }\left(\theta =\pi ,{k}_{\text{ex}}{R}_{\text{ex}}\right)\right|$ as the key optimization cost function. In contrast, for a high number of smart hybrid PZT/SVE cloaking layers $\left({N}_{\text{max}}\ge 11\right)$, a near-perfect scattering control can be achieved in the entire domain (i.e., in both forward and backward directions). Lastly, based on the above brief discussions on Figures 3, 4, 5, 6 and 7, the most interesting observation is perhaps the fact that in case of the relatively larger structural deflections associated with low to intermediate frequency range $\left({0<k}_{\text{ex}}{R}_{\text{ex }}\le 15\right),$ the type and number of viscoelastic damping interlayers (VE or SVE) appear to be the key factor in obtaining the best cloaking efficiency. On the other hand, in case of the relatively smaller structural deflections associated with high frequency range $\left({15<k}_{\text{ex}}{R}_{\text{ex }}\le 30\right),$ the presence of PZT actuator layers appears to be the dominant factor and the control action as well as the type of viscoelastic interlayers are not highly decisive.

**Figure 8:**
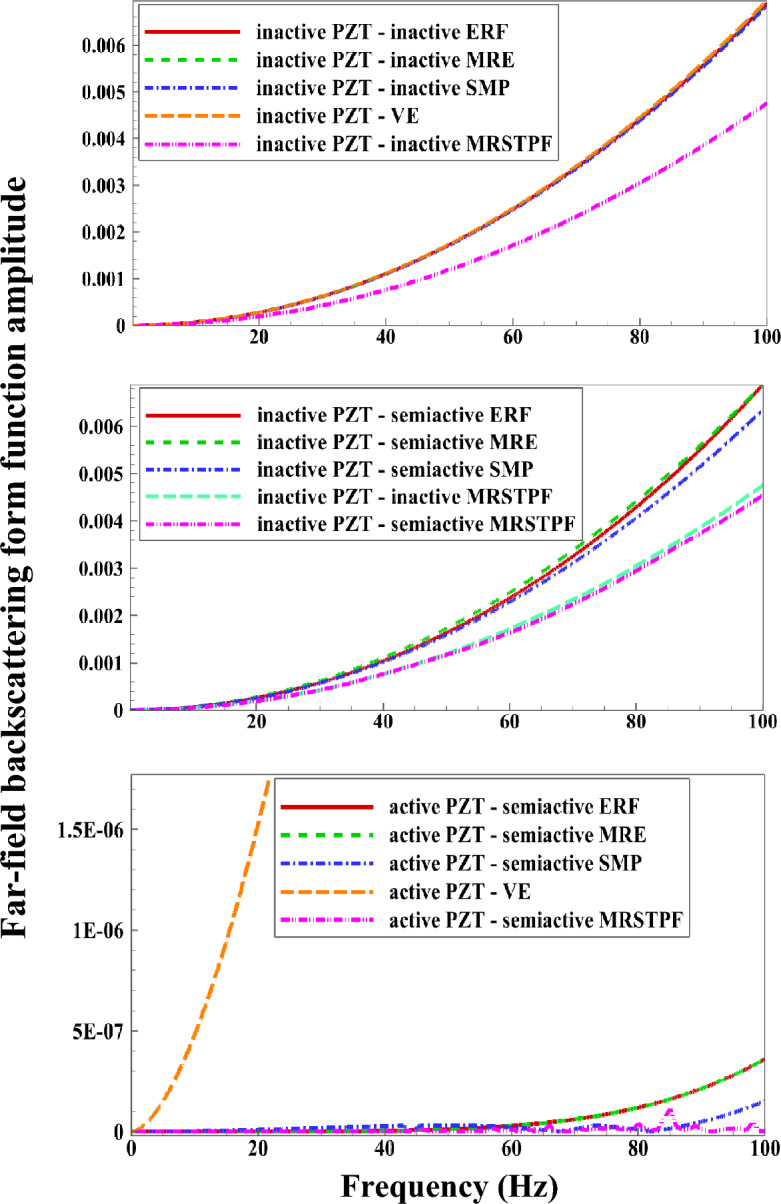
Low frequency far-field backscattering form function spectrums of the bimorph sandwich piezoelectric spherical shell cloaks for selected functional viscoelastic core materials and control configurations.

Lastly, the first subplot in Fig. 8 compares the low frequency backscattering form function amplitude spectrums in the far-field, $\left|{f}_{\infty }\right|,$ for the fully inactive thick-walled bimorph sandwich piezoelectric spherical shells (*N* = 3) for five selected functional viscoelastic core materials, namely, conventional viscoelastic material (VE), magnetorheological elastomer (MRE), shape memory polymer (SMP), electrorheological fluid (ERF), and magnetorheological shear thickening polishing fluid (MRSTPF). The input data for the complex loss and storage moduli of the MRSTPF calculations are directly taken from the experimentally-based low frequency $\left(0<f\le 100\text{Hz}\right)$ results provided in Fig. 9 of Ref^90^ Similarly, the second subplot in Fig. 8 compares $\left|{f}_{\infty }\right|$ for the thick-walled bimorph sandwich piezoelectric spherical shells (*N* = 3) with inactive PZT skin layers coupled with semiactive SVE (MRE,SMP,ERF,MRSTPF) core layers. Lastly, the third subplot in Fig. 8 compares $\left|{f}_{\infty }\right|$ for the thick-walled bimorph sandwich piezoelectric spherical shells (*N* = 3) with active PZT skin layers coupled with semiactive SVE (MRE,SMP,ERF,MRSTPF) core layers. Also shown (for comparison purposes) in the last subplot is the $\left|{f}_{\infty }\right|$ for the active bimorph PZT/VE/PZT spherical cloak. Comments very similar to the previous remarks on Figs. 3, 4, 5, 6 and 7 can readily be made. The most important distinction here is the noticeably higher effectiveness of both inactive and semi-active bimorph sandwich PZT/MRSTPF/PZT cloaks in comparison to the PZT/MRE/PZT, PZT/SMP/PZT, and PZT/ERF/PZT counterparts in the considered frequency band. In general, the notable advantage of the inactive bimorph sandwich PZT/MRSTPF/PZT cloak against the semiactive PZT/MRE/PZT, PZT/SMP/PZT, and PZT/ERF/PZT cloaks is evident in the second subplot of Fig. 8. This implies that comparable acoustic cloaking effects can be readily achieved without expenditure of any external energy only by utilization of inactive MRSTPF interlayers. Further improvements in the cloaking efficiency can evidently be attained through activation of the PZT constraining layers, as it is suggested in the third subplot of Fig. 8.

## Conclusions

Incident acoustic plane wave scattering cancellation from an arbitrarily thick multimorph concentric spherical shell cloak comprised of a finite sequence of perfectly interstacked alternating complementary PZT/SVE actuator layers is investigated. The acoustic cloaking performance of proposed configuration is systematically evaluated for four distinct classes of functional SVE core materials with tunable field-dependent rheological properties (i.e., MRE, SMP, ERF, and MRSTPF) in the framework of a multi-control hybrid active/semi-active control strategy. Extensive numerical simulations are carried out aiming at two key important questions, namely, how many and what type of viscoelastic interlayers are needed to achieve the best conceivable broadband acoustic invisibility? In this context, the inactive and active far-field backscattering form function amplitudes as well as the percent cloaking error ($\%\text{Err})$ spectrums are calculated for the smart hybrid bimorph piezoelectric $(N=3)$ as well as for the multimorph piezoelectric $(N=\text{3,7},\text{11,15,19,23,27,31})$ spherical shell transducers in a relatively broad range of frequencies. Furthermore, the backscattering form function spectrums of the hybrid smart bimorph PZT/SVE/PZT spherical shells in different control configurations are compared with those of a conventional PZT/VE shell in the low frequency range.

The numerical results reveal that the smart hybrid multimorph configurations with SMP and ERF core layers display a slightly better overall spectral cloaking performance compared to the configuration with MRE core layers, especially at low to intermediate dimensionless frequencies $\left({0<k}_{\text{ex}}{R}_{\text{ex }}\le 15\right)$. Also, the smart hybrid multimorph configurations show a markedly better overall spectral cloaking performance with respect to the configuration with VE core layers, especially at $\left({0<k}_{\text{ex}}{R}_{\text{ex }}\le 15\right)$. In particular, the calculated $\%\text{Err}$ for the smart hybrid PZT/VE and PZT/SVE multimorph piezoelectric spherical shells at intermediate to high incident wave frequencies markedly decrease with increasing the number of cloaking layers. Also, the overall cloaking performances of the smart multimorph PZT/VE and PZT/SVE configurations in terms of the calculated ${N}_{1\text{\%}}$ (or the minimum number of layers required for $\%\text{Err}<1\%$) are found to be very similar in the high dimensionless frequency range $\left({15<k}_{\text{ex}}{R}_{\text{ex }}\le 30\right),$ where the presence of PZT actuator layers appears to be the dominant factor on the frequency response characteristics (i.e., the type of viscoelastic interlayer is not determinative). In the low to intermediate dimensionless frequency range $\left({0<k}_{\text{ex}}{R}_{\text{ex }}\le 15\right)$, on the other hand, the type and number of viscoelastic (VE or SVE) damping interlayers seem to be the key elements, and the multimorph PZT/SVE cloaks demonstrate superior overall cloaking performance compared to the PZT/VE cloak, which are ranked in the associated critical regions as follows: PZT/SMP:$\%\text{Err}\left({N}_{\text{max}}=11\right)=0.0007;$ PZT/ERF:$\%\text{Err}\left({N}_{\text{max}}=11\right)=0.1793;$ PZT/MRE:$\%\text{Err}\left({N}_{\text{max}}=27\right)=0.0082;$ PZT/VE:$\%\text{Err}\left({N}_{\text{max}}=31\right)=4.51$. Moreover, the most interesting (benchmark) observation here is perhaps the fact that comparable acoustic cloaking effects may potentially be achieved (at least in the low frequency range) without expenditure of any external energy by employing the entirely inactive MRSTPF core layers, as an alternative to the semiactive or active SVE interlayers. In conclusion, the simulated results demonstrate that near-perfect three dimensional acoustic scattering control $(\%\text{Err}<1\%)$ with smooth and nearly flat frequency response characteristics over relatively wide frequency spectrums can effectively be accomplished in the entire physical domain (i.e., in both forward and backward directions) by means of multimorph spherical shell transducers with sufficient number of properly selected smart hybrid PZT/SVE cloaking layers. The outcome of proposed study can advantageously serve as the first step towards practical development and experimental implementation of future high performance smart underwater acoustic cloaking devices with expanded broadband near-perfect omnidirectional invisibility for three dimensional objects of diverse geometries. The upcoming research improvements could involve numerical simulation and experimental implementation of more sophisticated optimal multilayered smart hybrid acoustic cloaks that can function with minimum number of actuator layers in a much broader frequency band based on the state-of-the-art optimization schemes coupled with more advanced control techniques.

### Supplementary Information

Supplementary Information.

## Data Availability

The datasets used and/or analyzed during the current study are available from the corresponding author upon reasonable request.
