# Supplementary material for: A novel smart hybrid multimorph piezoelectric spherical shell cloak for broadband near-perfect underwater acoustic camouflage applications
Source: Sci Rep. 2024 Jun 7;14:13128. doi: 10.1038/s41598-024-63201-w (PMC11161647; doi:10.1038/s41598-024-63201-w)
Supplement: Supplementary file 1 — Supplementary Information. [file 41598_2024_63201_MOESM1_ESM.docx]

**Supplementary Material A. Modal coefficient matrices**

| $\mathbf{m}_{1,n}^{\left( 1,1 \right)}\left( \xi_{z,v}^{q_{v}^{z}} \right)=-2,$ | $\mathbf{m}_{1,n}^{\left( 2,1 \right)}\left( \xi_{z,v}^{q_{v}^{z}} \right)=\frac{C_{44}^{\mathrm{in}}}{\mu_{v}^{*}} ,$ | $\mathbf{m}_{1,n}^{\left( 1,2 \right)}\left( \xi_{z,v}^{q_{v}^{z}} \right)=\frac{\mu_{v}^{*}\left( l-2 \right)}{C_{44}^{\mathrm{in}}}-J_{z,v}^{k} ,$ |
| --- | --- | --- |
| $\mathbf{m}_{1,n}^{(2,2)}\left( \xi_{z,v}^{q_{v}^{z}} \right)= 1,$ | $\mathbf{m}_{2,0}^{\left( 1,1 \right)}(\xi_{z,v}^{q_{v}^{z}})=2\beta_{v}-1,$ | $\mathbf{m}_{2,0}^{\left( 1,2 \right)}(\xi_{z,v}^{q_{v}^{z}})=-\frac{2k_{1v}}{C_{44}^{\mathrm{in}}}-J_{z,v}^{k} ,$ |
| $\mathbf{m}_{2,0}^{\left( 2,1 \right)}(\xi_{z,v}^{q_{v}^{z}})=\frac{C_{44}^{\mathrm{in}}}{\lambda_{v}^{*}}+2\mu_{v}^{*} ,$ | $\mathbf{m}_{2,0}^{\left( 2,2 \right)}(\xi_{z,v}^{q_{v}^{z}})=-2\beta_{v} ,$ | $\mathbf{M}_{1,n}^{\left( 1,1 \right)}\left( \xi_{z,p}^{q_{p}^{z}} \right)=-2,$ |
| $\mathbf{M}_{1,n}^{\left( 1,2 \right)}\left( \xi_{z,p}^{q_{p}^{z}} \right)=\frac{C_{66}\left( l-2 \right)}{C_{44}^{\mathrm{in}}}-J_{z,p}^{k} ,$ | $\mathbf{M}_{1,n}^{\left( 2,1 \right)}\left( \xi_{z,p}^{q_{p}^{z}} \right)=\frac{C_{44}^{\mathrm{in}}}{\mu_{v}^{*}} ,$ | $\mathbf{M}_{1,n}^{\left( 2,2 \right)}\left( \xi_{z,p}^{q_{p}^{z}} \right)=1 ,$ |
| $\mathbf{m}_{2,n}^{\left( 1,1 \right)}\left( \xi_{z,v}^{q_{v}^{z}} \right)=2\beta_{v}-1,$ | $\mathbf{m}_{2,n}^{\left( 1,2 \right)}\left( \xi_{z,v}^{q_{v}^{z}} \right)=-l ,$ | $\mathbf{m}_{2,n}^{\left( 1,3 \right)}\left( \xi_{z,v}^{q_{v}^{z}} \right)=-\frac{k_{1v}l}{C_{44}^{\mathrm{in}}} ,$ |
| $\mathbf{m}_{2,n}^{\left( 1,4 \right)}\left( \xi_{z,v}^{q_{v}^{z}} \right)=-\frac{2k_{1v}}{C_{44}^{\mathrm{in}}}-J_{z,v}^{k} ,$ | $\mathbf{m}_{2,n}^{\left( 2,1 \right)}\left( \xi_{z,v}^{q_{v}^{z}} \right)=\beta_{v} ,$ | $\mathbf{m}_{2,n}^{\left( 2,2 \right)}\left( \xi_{z,v}^{q_{v}^{z}} \right)=-2 ,$ |
| $\mathbf{m}_{2,n}^{\left( 2,3 \right)}\left( \xi_{z,v}^{q_{v}^{z}} \right)=-\frac{k_{2v}l+2\mu_{v}^{*}}{C_{44}^{\mathrm{in}}}-J_{z,v}^{k} ,$ | $\mathbf{m}_{2,n}^{\left( 2,4 \right)}\left( \xi_{z,v}^{q_{v}^{z}} \right)=-\frac{k_{1v}}{C_{44}^{\mathrm{in}}} ,$ | $\mathbf{m}_{2,n}^{\left( 3,2 \right)}\left( \xi_{z,v}^{q_{v}^{z}} \right)=\frac{C_{44}^{\mathrm{in}}}{\mu_{v}^{*}} ,$ |
| $\mathbf{m}_{2,n}^{\left( 3,3 \right)}\left( \xi_{z,v}^{q_{v}^{z}} \right)=1,$ | $\mathbf{m}_{2,n}^{\left( 3,4 \right)}\left( \xi_{z,v}^{q_{v}^{z}} \right)=1,$ | $\mathbf{m}_{2,n}^{\left( 4,1 \right)}\left( \xi_{z,v}^{q_{v}^{z}} \right)=\frac{C_{44}^{\mathrm{in}}}{\lambda_{v}^{*}}+2\mu_{v}^{*} ,$ |
| $\mathbf{m}_{2,n}^{\left( 4,3 \right)}\left( \xi_{z,v}^{q_{v}^{z}} \right)=-\beta_{v}l ,$ | $\mathbf{m}_{2,n}^{\left( 4,4 \right)}\left( \xi_{z,v}^{q_{v}^{z}} \right)=-2\beta_{v} ,$ | $\mathbf{M}_{2,0}^{\left( 1,1 \right)}\left( \xi_{z,p}^{q_{p}^{z}} \right)=2\beta-1,$ |
| $\mathbf{M}_{2,0}^{\left( 1,2 \right)}\left( \xi_{z,p}^{q_{p}^{z}} \right)=-\frac{2k_{1}}{C_{44}^{\mathrm{in}}}-J_{z,p}^{k},$ | $\mathbf{M}_{2,0}^{\left( 1,3 \right)}\left( \xi_{z,p}^{q_{p}^{z}} \right)=\frac{2\gamma e_{33}^{\mathrm{in}}}{C_{44}^{in}},$ | $\mathbf{M}_{2,0}^{\left( 2,1 \right)}\left( \xi_{z,p}^{q_{p}^{z}} \right)=\frac{C_{44}^{\mathrm{in}}\varepsilon_{3}}{\alpha},$ |
| $\mathbf{M}_{2,0}^{\left( 2,2 \right)}\left( \xi_{z,p}^{q_{p}^{z}} \right)=-2\beta,$ | $\mathbf{M}_{2,0}^{\left( 2,3 \right)}\left( \xi_{z,p}^{q_{p}^{z}} \right)=\frac{e_{33}e_{33}^{\mathrm{in}}}{\alpha},$ | $\mathbf{M}_{2,0}^{\left( 3,3 \right)}\left( \xi_{z,p}^{q_{p}^{z}} \right)=-1,$ |
| $\mathbf{M}_{2,0}^{\left( 3,4 \right)}\left( \xi_{z,p}^{q_{p}^{z}} \right)=-\frac{k_{3}l}{\varepsilon_{33}^{\mathrm{in}}} ,$ | $\mathbf{M}_{2,0}^{\left( 4,1 \right)}\left( \xi_{z,p}^{q_{p}^{z}} \right)=\frac{e_{33}C_{44}^{\mathrm{in}}\varepsilon_{33}^{\mathrm{in}}}{{\alpha e}_{33}^{\mathrm{in}}},$ | $\mathbf{M}_{2,0}^{\left( 4,2 \right)}\left( \xi_{z,p}^{q_{p}^{z}} \right)=-\frac{2\gamma\varepsilon_{33}^{\mathrm{in}}}{e_{33}^{\mathrm{in}}},$ |
| $\mathbf{M}_{2,0}^{\left( 4,3 \right)}\left( \xi_{z,p}^{q_{p}^{z}} \right)=-\frac{C_{33}\varepsilon_{33}^{\mathrm{in}}}{\alpha} ,$ | $\mathbf{M}_{2,n}^{\left( 1,1 \right)}\left( \xi_{z,p}^{q_{p}^{z}} \right)=2\beta-1,$ | $\mathbf{M}_{2,n}^{\left( 1,2 \right)}\left( \xi_{z,p}^{q_{p}^{z}} \right)=-l,$ |
| $\mathbf{M}_{2,n}^{\left( 1,3 \right)}\left( \xi_{z,p}^{q_{p}^{z}} \right)=\frac{{-k}_{1}l}{C_{44}^{\mathrm{in}}} ,$ | $\mathbf{M}_{2,n}^{\left( 1,4 \right)}\left( \xi_{z,p}^{q_{p}^{z}} \right)=-\frac{2k_{1}}{C_{44}^{\mathrm{in}}}-J_{z,p}^{k} ,$ | $\mathbf{M}_{2,n}^{\left( 1,5 \right)}\left( \xi_{z,p}^{q_{p}^{z}} \right)=\frac{2\gamma e_{33}^{\mathrm{in}}}{C_{44}^{\mathrm{in}}},$ |
| $\mathbf{M}_{2,n}^{\left( 2,1 \right)}\left( \xi_{z,p}^{q_{p}^{z}} \right)=\beta,$ | $\mathbf{M}_{2,n}^{\left( 2,2 \right)}\left( \xi_{z,p}^{q_{p}^{z}} \right)=-2,$ | $\mathbf{M}_{2,n}^{\left( 2,3 \right)}\left( \xi_{z,p}^{q_{p}^{z}} \right)=-\frac{k_{2}l+2C_{66}}{C_{44}^{\mathrm{in}}}-J_{z,p}^{k} ,$ |
| $\mathbf{M}_{2,n}^{\left( 2,4 \right)}\left( \xi_{z,p}^{q_{p}^{z}} \right)=-\frac{k_{1}}{C_{44}^{\mathrm{in}}} ,$ | $\mathbf{M}_{2,n}^{\left( 2,5 \right)}\left( \xi_{z,p}^{q_{p}^{z}} \right)=\frac{\gamma e_{33}^{\mathrm{in}}}{C_{44}^{\mathrm{in}}},$ | $\mathbf{M}_{2,n}^{\left( 3,2 \right)}\left( \xi_{z,p}^{q_{p}^{z}} \right)=\frac{C_{44}^{\mathrm{in}}}{C_{44}} ,$ |
| $\mathbf{M}_{2,n}^{\left( 3,3 \right)}\left( \xi_{z,p}^{q_{p}^{z}} \right)=1,$ | $\mathbf{M}_{2,n}^{\left( 3,4 \right)}\left( \xi_{z,p}^{q_{p}^{z}} \right)=1,$ | $\mathbf{M}_{2,n}^{\left( 3,6 \right)}\left( \xi_{z,p}^{q_{p}^{z}} \right)=\frac{e_{15}e_{33}^{\mathrm{in}}}{C_{44}\varepsilon_{33}^{\mathrm{in}}} ,$ |
| $\mathbf{M}_{2,n}^{\left( 4,1 \right)}\left( \xi_{z,p}^{q_{p}^{z}} \right)=\frac{C_{44}^{\mathrm{in}}\varepsilon_{3}}{\alpha},$ | $\mathbf{M}_{2,n}^{\left( 4,3 \right)}\left( \xi_{z,p}^{q_{p}^{z}} \right)=-\beta l,$ | $\mathbf{M}_{2,n}^{\left( 4,4 \right)}\left( \xi_{z,p}^{q_{p}^{z}} \right)=-2\beta,$ |
| $\mathbf{M}_{2,n}^{\left( 4,5 \right)}\left( \xi_{z,p}^{q_{p}^{z}} \right)=\frac{e_{33}e_{33}^{\mathrm{in}}}{\alpha} ,$ | $\mathbf{M}_{2,n}^{\left( 5,2 \right)}\left( \xi_{z,p}^{q_{p}^{z}} \right)=-\frac{e_{15}C_{44}^{\mathrm{in}}l}{e_{33}^{in}C_{44}} ,$ | $\mathbf{M}_{2,n}^{\left( 5,5 \right)}\left( \xi_{z,p}^{q_{p}^{z}} \right)=-1,$ |
| $\mathbf{M}_{2,n}^{\left( 5,6 \right)}\left( \xi_{z,p}^{q_{p}^{z}} \right)=-\frac{k_{3}l}{\varepsilon_{33}^{\mathrm{in}}}$ | $\mathbf{M}_{2,n}^{\left( 6,1 \right)}\left( \xi_{z,p}^{q_{p}^{z}} \right)=\frac{e_{33}C_{44}^{\mathrm{in}}\varepsilon_{33}^{\mathrm{in}}}{{\alpha e}_{33}^{\mathrm{in}}} ,$ | $\mathbf{M}_{2,n}^{\left( 6,3 \right)}\left( \xi_{z,p}^{q_{p}^{z}} \right)=-\frac{\gamma\varepsilon_{33}^{\mathrm{in}}l}{e_{33}^{\mathrm{in}}},$ |
| $\mathbf{M}_{2,n}^{\left( 6,4 \right)}\left( \xi_{z,p}^{q_{p}^{z}} \right)=-\frac{2\gamma\varepsilon_{33}^{\mathrm{in}}}{e_{33}^{\mathrm{in}}} ,$ | $\mathbf{M}_{2,n}^{\left( 6,5 \right)}\left( \xi_{z,p}^{q_{p}^{z}} \right)=-\frac{C_{33}\varepsilon_{33}^{\mathrm{in}}}{\alpha} ,$ |  |

where $l=n(n+1)$, $J_{z,v}^{k}=(\frac{\rho_{v}}{\rho_{p}^{\mathrm{in}}})\left( \frac{\xi_{z,v}^{q_{v}^{z}}}{R_{\mathrm{in}}} \right)^{2}\Omega^{2}e^{2\xi_{z,v}^{k}}$, $J_{z,p}^{k}=(\frac{\rho_{p}}{\rho_{p}^{\mathrm{in}}})\left( \frac{\xi_{z,p}^{q_{p}^{z}}}{R_{\mathrm{in}}} \right)^{2}\Omega^{2}e^{2\xi_{z,p}^{k}}$, $\Omega^{2}=\omega^{2}R_{\mathrm{in}}^{2}\rho_{p}^{\mathrm{in}}/C_{44}^{\mathrm{in}}$ is the dimensionless frequency, ($\xi_{z,v}^{q_{v}^{z}}$,$\xi_{z,p}^{q_{p}^{z}}$) signify the external radii of each SVE or PZT layer, and $\rho_{p}^{\mathrm{in}}$ is the piezoelectric mass density at $r=R_{\mathrm{in}}$, and

| $\alpha=C_{33}\varepsilon_{33}+e_{33}^{2},$  $\beta={(C_{13}\varepsilon_{33}+e_{31}e_{33})}/\alpha,$  $\gamma={(C_{13}e_{33}-C_{33}e_{31})}/\alpha,$  $\beta_{v}=\lambda_{v}^{*}/(\lambda_{v}^{*}+2\mu_{v}^{*}),$  $k_{1v}=2\lambda_{v}^{*}\beta_{v}-2(\lambda_{v}^{*}+\mu_{v}^{*}),$ | $k_{1}=2\left( C_{13}\beta+e_{31}\gamma\right)-\left( C_{11}+C_{12} \right),$  $k_{2}=(k_{1}/2)-C_{66},$  $k_{3}=\varepsilon_{11}+\left( e_{15}^{2}/C_{44} \right),$  $k_{2v}=\frac{k_{1v}}{2}-\mu_{v}^{*}.$ |  |
| --- | --- | --- |

**Supplementary Material B. Viscoelastic models**

**Havriliak-Negami (HN).** Among the various classical viscoelastic (VE) models, one successful description for the frequency dependence of the complex modulus, $G_{v}^{*}=G^{'}\left( \omega\right)+iG^{''}\left( \omega\right),$ of polymers in the viscoelastic glass transition region is provided by the so called Havriliak-Negami (HN) [91]. Based on the HN model, the real and imaginary parts of the complex modulus are given as [92]:

|  | $G^{'}\left( \omega\right)=G_{\infty}+\frac{\left( G_{0}-G_{\infty} \right)\cos\left( \beta\theta\right)}{\left[ 1+2\omega^{\alpha}\tau^{\alpha}\cos\gamma+\omega^{2\alpha}\tau^{2\alpha} \right]^{\frac{\beta}{2}}},$  $G^{''}\left( \omega\right)=\frac{\left( G_{\infty}-G_{0} \right)\sin\left( \beta\theta\right)}{\left[ 1+2\omega^{\alpha}\tau^{\alpha}\cos\gamma+\omega^{2\alpha}\tau^{2\alpha} \right]^{\frac{\beta}{2}}} ,$ | (B-1) |
| --- | --- | --- |

where $\rho_{v} =1096(kg/m^{3}),$ $\nu_{v}=0.49, \theta\left( \omega\right)=\tan^{-1} \frac{\omega^{\alpha}\tau^{\alpha}sin\gamma}{1+\omega^{\alpha}\tau^{\alpha}cos\gamma} ,$ $\gamma=\alpha\pi/2,$ $\tau=0.1702\sec$ is the relaxation time, the dimensionless parameter $\left( 0 < \alpha=0.4941 <1 \right)$ regulates the relaxation range, the dimensionless parameter (0 < $\beta=0.1356$ <1) controls the relaxation asymmetry, and $G_{0}=5.019\times{10}^{7}(dynes/cm^{2})$ and $G_{\infty}=0.8089\times{10}^{10}(dynes/cm^{2})$ signify the limiting shear modulus at low and high frequencies, respectively.

**Magnetorheological Elastomer (MRE).** The real and imaginary parts of the magnetic field-dependent complex shear modulus of MR elastomer, $G_{v}^{*}=G^{'}\left( B \right)+iG^{''}\left( B \right),$ in the pre-yield regime based on the linear viscoelastic theory has been experimentally estimated in terms of the intensity of magnetic field ($0\leq B\leq500$G) by the following second-order polynomial function [93,94]**:**

|  | $G^{'}\left( B \right)=-3.3691B^{2}+4997.5 B+873000,$  $G^{''}\left( B \right)=-0.9B^{2}+812.4 B+185500 ,$ | (B-2) |
| --- | --- | --- |

with $\rho_{v} =3500(kg/m^{3}),$ and $\nu_{v}=0.49$.

**Shape Memory Polymer (SMP).** By adopting 2S2P1D (i.e., abbreviation for two Springs, two Parabolic creep element and one Dashpot) viscoelastic model, the experimentally validated damping property of tBA/PEGDMA SMP in a wide range of temperature and frequency can be estimated as [95,96]:

|  | $E_{v}^{*}\left( \omega,T \right)=E_{0}+\frac{E_{\infty}-E_{0}}{1+\gamma\left( j\omega\tau\right)^{-k}+\left( j\omega\tau\right)^{-h}+\left( j\omega\beta\tau\right)^{-1}} ,$ | (B-3) |
| --- | --- | --- |

where $E_{v}^{*}$ is the complex modulus of SMP polymer, $(E_{0}=0.67 \mathrm{MPa} {,E}_{\infty}=2211 \mathrm{MPa})$ respectively refer to modulus of the rubber at the zero and infinity frequency limits, $\gamma=1.68$, $\beta=3.8\times{10}^{4}$, $0 <k=0.16<h=0.79<1$ are constant exponents. Also, based on the so called temperature-time superposition principle, the characteristic time for ($22\leq T\leq130℃$) is estimated as $\tau\left( T \right)=a_{T}\left( T \right)\tau_{0},$ where the shift factor $a_{T}\left( T \right)$ is determined from $\log\left( a_{T}\left( T \right) \right)={-C_{1}^{0}(T-T_{0})}/{(C_{2}^{0}+\left( T-T_{0} \right))} ,$ in which $T_{0}$ is the reference temperature and $\tau_{0}=\tau\left( T_{0} \right)=0.61$,$C_{1}^{0}$=10.87, $C_{2}^{0}=32.57$, and with $\rho_{v} =990.4 (kg/m^{3}),$ $\nu_{v}=0.37$.

**Electrorheological Fluid (ERF).** The ERF-core material adopted here is supposed to obey the first order Kelvin-Voigt viscoelastic model in the pre-yield regime ($0\leq E\leq5$kV/mm), with the following form of experimentally extracted input parameters for the (quadratic) electric field dependency of the real and imaginary parts of the complex shear modulus, $G_{v}^{*}=G^{'}\left( E \right)+iG^{''}\left( E \right),$in the form [97]:

| $G_{c}^{'}=0.0103E^{2}+87.9024E+62162.8751$,  $G_{c}^{''}=0.0109E^{2}+75.6028E+28244.1486.$ | (B-4) |
| --- | --- |

with $\rho_{v} =1400(kg/m^{3}),$ and $\nu_{v}=0.49$.
